# Oligodendroglial myelination requires astrocyte-derived lipids

**DOI:** 10.1371/journal.pbio.1002605

**Published:** 2017-05-26

**Authors:** Nutabi Camargo, Andrea Goudriaan, Anne-Lieke F. van Deijk, Willem M. Otte, Jos F. Brouwers, Hans Lodder, David H. Gutmann, Klaus-Armin Nave, Rick M. Dijkhuizen, Huibert D. Mansvelder, Roman Chrast, August B. Smit, Mark H. G. Verheijen

**Affiliations:** 1 Department of Molecular and Cellular Neurobiology, Center for Neurogenomics and Cognitive Research, Amsterdam Neuroscience, VU University Amsterdam, Amsterdam, the Netherlands; 2 Biomedical MR Imaging and Spectroscopy group, Center for Image Sciences, University Medical Center Utrecht, Utrecht, the Netherlands; 3 Department of Pediatric Neurology, Brain Center Rudolf Magnus, University Medical Center Utrecht, Utrecht, the Netherlands; 4 Department of Biochemistry and Cell Biology, Faculty of Veterinary Medicine, Utrecht University, the Netherlands; 5 Department of Integrative Neurophysiology, Center for Neurogenomics and Cognitive Research, Amsterdam Neuroscience, VU University Amsterdam, Amsterdam, the Netherlands; 6 Department of Neurology, Washington University School of Medicine, St. Louis, Missouri, United States of America; 7 Max-Planck-Institute of Experimental Medicine, Department of Neurogenetics, Goettingen, Germany; 8 Department of Neuroscience and Department of Clinical Neuroscience, Karolinska Institutet, Stockholm, Sweden; Max-Planck-Institut fur experimentelle Medizin, GERMANY

## Abstract

In the vertebrate nervous system, myelination of axons for rapid impulse propagation requires the synthesis of large amounts of lipids and proteins by oligodendrocytes and Schwann cells. Myelin membranes are thought to be cell-autonomously assembled by these axon-associated glial cells. Here, we report the surprising finding that in normal brain development, a substantial fraction of the lipids incorporated into central nervous system (CNS) myelin are contributed by astrocytes. The oligodendrocyte-specific inactivation of sterol regulatory element-binding protein (SREBP) cleavage-activating protein (SCAP), an essential coactivator of the transcription factor SREBP and thus of lipid biosynthesis, resulted in significantly retarded CNS myelination; however, myelin appeared normal at 3 months of age. Importantly, embryonic deletion of the same gene in astrocytes, or in astrocytes and oligodendrocytes, caused a persistent hypomyelination, as did deletion from astrocytes during postnatal development. Moreover, when astroglial lipid synthesis was inhibited, oligodendrocytes began incorporating circulating lipids into myelin membranes. Indeed, a lipid-enriched diet was sufficient to rescue hypomyelination in these conditional mouse mutants. We conclude that lipid synthesis by oligodendrocytes is heavily supplemented by astrocytes in vivo and that horizontal lipid flux is a major feature of normal brain development and myelination.

## Abstract 2

The formation of myelin membrane by oligodendrocytes in the brain is dependent on extracellular lipids derived from astrocytes and from diet.

## Author summary

The myelin membrane is a highly specialized plasma membrane that enwraps axons, acts as an insulator, and is thus important for fast conduction of action potentials. It is thereby critical for proper functioning of the nervous system. Myelin in the central nervous system is synthesized by oligodendrocytes. Because myelin has a high lipid content, it is vulnerable to lipid metabolism disorders. It is unknown whether extracellular lipids also contribute to myelination by oligodendrocytes under healthy conditions, and if they do, what the origin of these lipids would be.

Here, we show that formation of myelin membrane in mice does not only need lipid synthesis by oligodendrocytes, but also requires extracellular lipids provided by astrocytes. Indeed, when lipid synthesis was inactivated in either oligodendrocytes or astrocytes, myelin membrane synthesis was reduced. However, when lipid synthesis was inactivated in both cell types, myelin membrane synthesis was virtually absent. Furthermore, when lipid synthesis was inactivated in astrocytes, oligodendrocytes bypassed this deficiency by using dietary lipids for myelin membrane synthesis. Furthermore, a high-fat diet could promote myelin synthesis. We conclude that extracellular lipids, either provided by astrocytes or in the diet, contribute to myelination by oligodendrocytes during normal brain development.

## Introduction

Myelin membrane integrity is critical for proper functioning of the nervous system. Myelin acts as an insulator by increasing the electrical resistance across the cell membrane and by decreasing membrane capacitance, thereby ensuring the fast conduction of action potentials between nodes of Ranvier over long distances[1,2]. Myelin is a specialized membrane organelle synthesized by Schwann cells (SC) in the peripheral nervous system (PNS) and by oligodendrocytes in the central nervous system (CNS) [3]. A prominent biochemical characteristic of myelin is its high lipid-to-protein ratio. Lipids account for at least 70% of the dry weight of the myelin membrane[4], which is twice that of other plasma membranes[5]. The high lipid content of the myelin membrane makes it vulnerable for lipid metabolism disorders[5] and makes lipid availability rate-limiting for myelination. Accordingly, genetic impairment of endogenous lipid synthesis in SC interferes with the acute phase of PNS myelination[6]. Interestingly, uptake of extracellular lipids by these cells partially rescues myelination over time[6]. Similarly, mice carrying an oligodendrocyte-specific deletion of squalene synthase (SQS), an enzyme required for cholesterol synthesis, have CNS hypomyelination, but this marks a delay, and myelination becomes nearly normal at 3 months[7]. It is unknown whether extracellular lipids also contribute to myelination by oligodendrocytes in the CNS under healthy conditions, and, if they do, what the origin of these lipids would be.

The CNS is classically viewed as being largely autonomous in lipid metabolism since it is shielded from lipids in the circulation by the blood–brain barrier[8,9]. One cellular source of lipid synthesis and secretion is the astrocyte[10–17], and in vitro studies have shown that astrocytes are able to promote myelination in neuron-oligodendrocyte co-cultures[18–20]. Recently, we found that cholesterol and fatty acid synthesis in astrocytes relies on sterol regulatory element binding proteins (SREBPs)[11]. SREBPs, consisting of SREBP-1a, SREBP-1c, and SREBP-2, belong to the family of basic helix–loop–helix leucine zipper (bHLH-Zip) transcription factors that govern the transcriptional activation of genes involved in fatty acid and cholesterol metabolism[21] and are posttranslationally activated by the sterol sensor SREBP cleavage-activating protein (SCAP)[22]. The recent demonstration that SREBPs are regulated by mTORC1, a signaling complex important for both PNS and CNS myelination [23,24], is consistent with an important role of the SCAP–SREBP pathway in both SC [6] and oligodendrocytes.

Here, we used glial cell-restricted inactivation of SCAP-SREBP–mediated lipid biogenesis to determine the individual role of oligodendrocyte and astrocyte lipid metabolism in CNS myelination. We found that myelin membrane formation not only builds on oligodendrocyte endogenous lipid synthesis, as generally thought, but also critically depends on extracellular lipids provided by astrocytes.

## Results

### SCAP deletion in oligodendrocytes interferes with the acute phase of myelination

**Fig 1:**
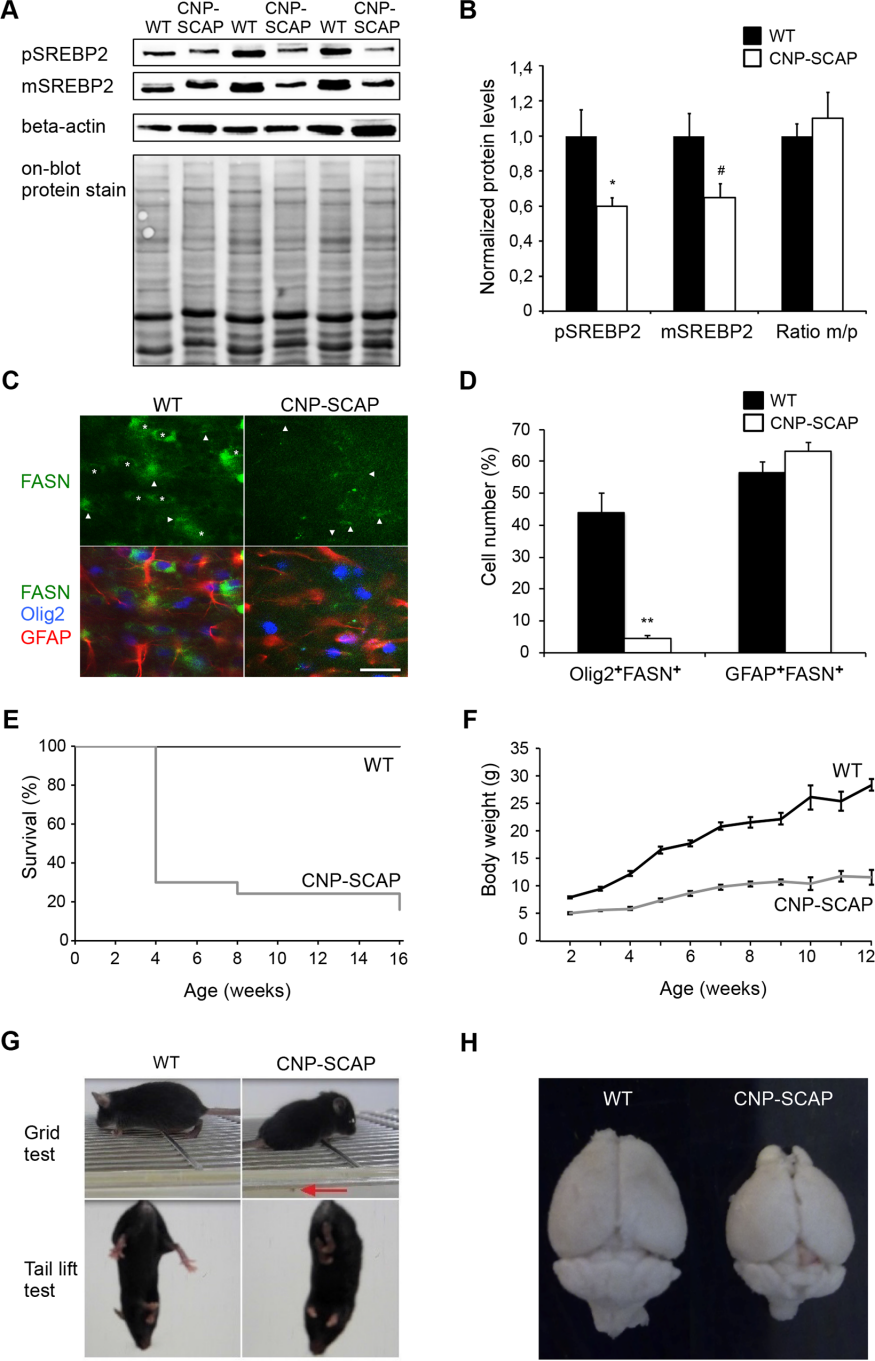
Conditional inactivation of SREBP cleavage-activating protein (SCAP) in oligodendrocytes reduces lipogenic gene expression and causes motor control defects and reduced survival. **A)** Protein levels of precursor sterol regulatory element-binding protein 2 (SREBP2) and mature (processed) SREBP2 were determined by immunoblotting of total extracts of the spinal cord of wild-type (WT) and CNP-SCAP animals at P20 (*n* = 3). Detection of mature and precursor SREBP2 was performed using different exposure times, and representative pictures are shown. Detection of beta-actin and on-blot protein stain was used to control for equal loading. **B)** Histogram shows quantification of precursor and mature SREBP2 protein levels after correction for equal loading (using on-blot stain) and subsequent normalization to WT levels in which the WT levels were set to 1, and the ratio between mature/precursor SREBP in which the ratio for WT was set to 1. All data are presented as mean levels ± SEM (*t* test: *p < 0.05, #*p* = 0.059). **C)** Expression of fatty acid synthase (FASN, green) in oligodendrocytes (Olig2, blue) and astrocytes (glial fibrillary acidic protein [GFAP], red) of WT or CNP-SCAP mutant mice at P20. Asterisks denote oligodendrocytes with FASN expression; arrowheads denote astrocytes with FASN expression (scale bar, 25 μm). **D)** Number of FASN-positive oligodendrocytes (Olig2^+^FASN^+^) and FASN-positive astrocytes (GFAP^+^FASN^+^) in WT and CNP-SCAP mice (*n* = 3). The values are provided as the percentage of the total number of oligodendrocytes (Olig2+ cells) or astrocytes (GFAP^+^ cells). Data are presented as mean ± SEM. **p < 0.01 using *t* test. **E)** Kaplan-Meier survival plot showing a strongly reduced life span of CNP-SCAP mice compared to age-matched WT mice. **F)** Body weight development of WT and CNP-SCAP mice over a 3-month period. Shown are the mean and SEM. **G)** At a grid test, CNP-SCAP mice showed limb ataxia, causing frequent slips of the hind limbs (red arrow) or front limbs. Mutant mice showed an abnormal reaction when tail lifted; they attempted to clasp their hind limbs and clench the toes of their rear feet. **H)** CNP-SCAP brains compared to control littermates at P28. The numeric data underlying Fig 1B, D, E and F can be found in S1 Data.

To inactivate lipid biosynthesis in oligodendrocytes, we crossed SCAP-floxed mice[22] with mice expressing Cre recombinase specifically in oligodendrocytes and SCs (CNP-Cre)[25]. CNP-cre/SCAPloxP/loxP mutant mice, referred to as CNP-SCAP mice in the following, were born at normal Mendelian ratios and were indistinguishable from controls at birth. SCAP is required for the processing of SREBPs into active transcription factors[22]. Accordingly, in CNP-SCAP animals, we detected reduced levels of cleaved (mature) SREBP2 proteins at P20 in spinal cord (where oligodendrocytes form a large cell population) (Fig 1A and 1B). In addition, SREBP2 precursor levels were strongly reduced (Fig 1A and 1B), which is consistent with previous observations that SCAP also regulates the expression of the SREBP genes[6,22]. The residual detectable SREBP2 protein in CNP-SCAP mice likely comes from other cell types, predominantly astrocytes, which are active in lipid metabolism[11,17,25]. As such, white matter expression of fatty acid synthase (FASN), a SREBP target gene [21], was observed in oligodendrocytes and strongly reduced in CNP-SCAP mutants (Fig 1C and 1D). Low FASN expression was observed in astrocytes and was unaffected in CNP-SCAP mutants (Fig 1C and 1D). No expression of FASN was observed in neurons in the cortex or hippocampus (S1 Fig). CNP-SCAP mice show reduced survival, probably caused by lethal seizures, with the most critical phase around weaning (weeks 2–4) (Fig 1E), and reduced weight gain (Fig 1F). Moreover, CNP-SCAP mice exhibit tremors and an unsteady gait after postnatal week 2 (Fig 1G), as well as microcephaly (Fig 1H).

**Fig 2:**
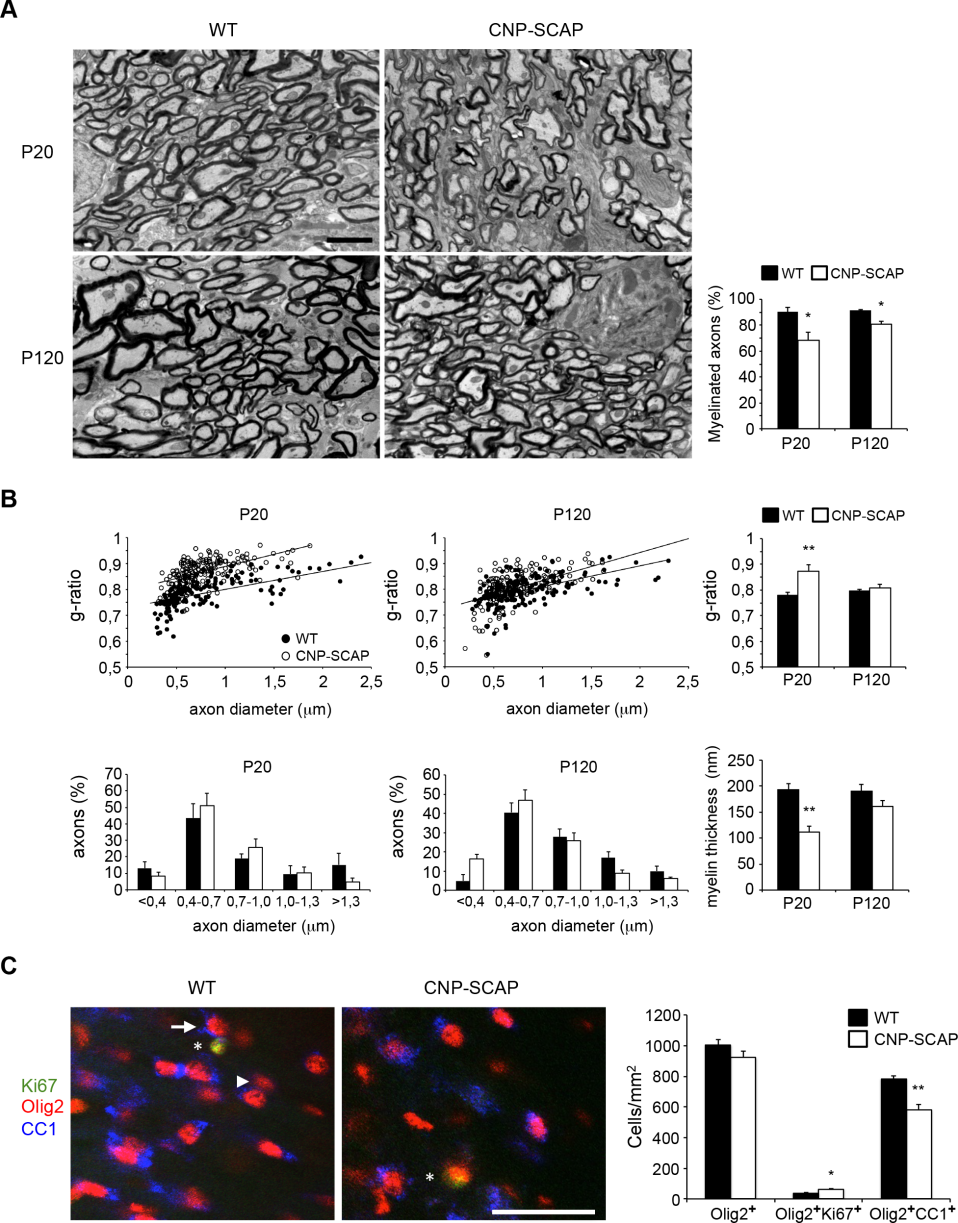
Reduced myelination in the central nervous system (CNS) of CNP-SREBP cleavage-activating protein (SCAP) mutant mice. **A)** Electron microscopic analysis of optic nerve myelin in cross-sections of either wild-type (WT) or CNP-SCAP mice at depicted time points (scale bar, 2 μm). Bar graph shows the percentage of axons that is myelinated. **B)** Morphometric analysis of axons in optic nerves of WT and CNP-SCAP mice, showing g-ratio (axon diameter/myelinated fiber diameter), axonal size distribution (both myelinated and nonmyelinated axons), and myelin membrane thickness at P20 and P120. At P20, the relation axon diameter (x) and g-ratio (y) was y = 7E − 05x + 0.7312 for WT and y = 9E − 05x + 0.796 for CNP-SCAP, with coefficients of determination R^2^ = 0.45013 (WT) and 0.28818 (CNP-SCAP). At P120: y = 8E − 05x + 0.731 (WT); y = 0.0001x + 0.7253 (CNP-SCAP), R^2^ = 0.2957 (WT) and 0.27524 (CNP-SCAP). **C)** Expression of postmitotic marker CC1 (blue) or proliferation marker Ki67 (green) in oligodendrocytes (Olig2, red) of WT or CNP-SCAP mutant mice in the corpus callosum at P20. The arrow and arrowhead denote examples of oligodendrocytes, respectively, with or without CC1 expression. The asterisks denote an oligodendrocyte with Ki67 expression (scale bar, 40 μm). The bar graph shows the density of the total number of oligodendrocytes (Olig2^+^ cells), immature oligodendrocytes (Olig2^+^Ki67^+^ cells), and postmitotic mature oligodendrocytes (Olig2^+^CC1^+^ cells) in WT and CNP-SCAP mice. Data are presented as mean ± SEM. * = *p* < 0.05 ** = *p* < 0.01 using *t* test, *n* = 3–4. The numeric data can be found in S1 Data.

Electron microscopy (EM) demonstrated that CNP-SCAP optic nerves were hypomyelinated at P20 and appeared normal at P120, although still mildly hypomyelinated (Fig 2A). G-ratio measurements of myelinated fibers confirmed that hypomyelination of the optic nerve was severe at P20 and restored to almost normal levels by P120 (Fig 2B). Axon diameter distribution was not significantly affected (Fig 2B). Accordingly, myelin membrane thickness of CNP-SCAP mice was thinner at P20 and improved at P120 (Fig 2B). The quantification of the number of Olig2^+^ cells (P20; Fig 2C) showed that oligodendrocyte-specific ablation of SCAP had no effect on oligodendrocyte precursor cell (OPC)/oligodendrocyte cell numbers, and proliferation of oligodendrocyte lineage cells (Ki67^+^Olig2^+^ cells) was slightly increased. The quantification of CC1^+^Olig2^+^ mature oligodendrocytes revealed lower numbers in CNP-SCAP mutants (Fig 2C).

**Fig 3:**
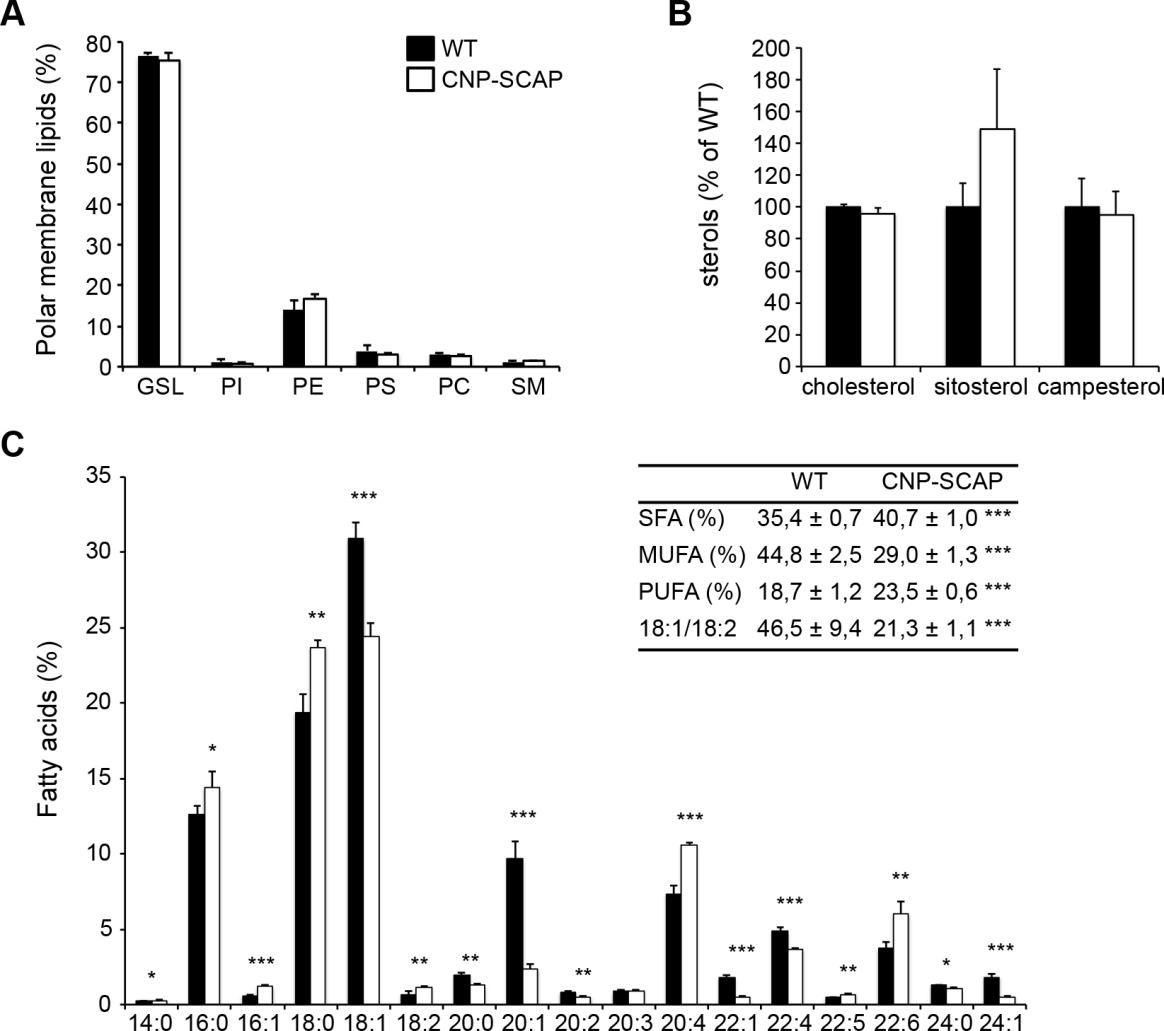
Myelin from CNP-SREBP cleavage-activating protein (SCAP) brains shows changes in fatty acid composition. Lipid extracts of purified myelin of wild-type (WT) and CNP-SCAP brains at P56 were analyzed using liquid chromatography and mass spectrometry. **A)** Polar membrane lipid concentration and **B)** sterol concentration per protein amount in CNP-SCAP compared to WT myelin. GSL, glycosphingolipid; PI, phosphatidyl inositol; PE, phosphatidyl ethanolamine; PS, phosphatidyl serine; PC, phosphatidyl choline; SM, sphingomyelin. **C)** Fatty acid profile of phospholipids from purified myelin with the amount of different fatty acids species as percentage of the total amount. Fatty acid species are depicted as “y:z,” with “y” giving the length of the fatty acid molecules and “z” the number of double bonds. Insert: depicted are proportions of saturated fatty acids (SFA), monounsaturated fatty acids (MUFA), polyunsaturated fatty acids (PUFA), and the ratio of 18:1/18:2. Data are presented as mean percentage of WT ± SD. *t* tests: * = *p* < 0.05; ** = *p* < 0.01; *** = *p* < 0.001, *n* = 5. The numeric data can be found in S1 Data.

Next, we determined the effect of SCAP deletion on myelin lipid composition. Lipid analysis of purified myelin of CNP-SCAP adult brains (P56) demonstrated no changes in phospholipid classes (Fig 3A) or sterols (Fig 3B). The fatty acid composition of phospholipids in mutant myelin was significantly shifted from monounsaturated fatty acids towards polyunsaturated fatty acids, which was also visible in a decrease in the ratio of 18:1/18:2 (Fig 3C). CNP-SCAP mutant myelin contained more polyunsaturated fatty acids and had higher levels of the essential fatty acid C18:2, which is consistent with an increased uptake of fatty acids from external sources[5,6].

Taken together, compromised lipid metabolism in oligodendrocytes leads to a severe developmental delay in myelin synthesis, accompanied by a compensatory increase in uptake of fatty acids from external sources and a largely improved phenotype in adult mice. This raises the question whether other cell types, in particular astrocytes, represent suppliers of lipids for lipogenesis-deficient oligodendrocytes.

### CNS hypomyelination in astrocyte SCAP mutants

**Fig 4:**
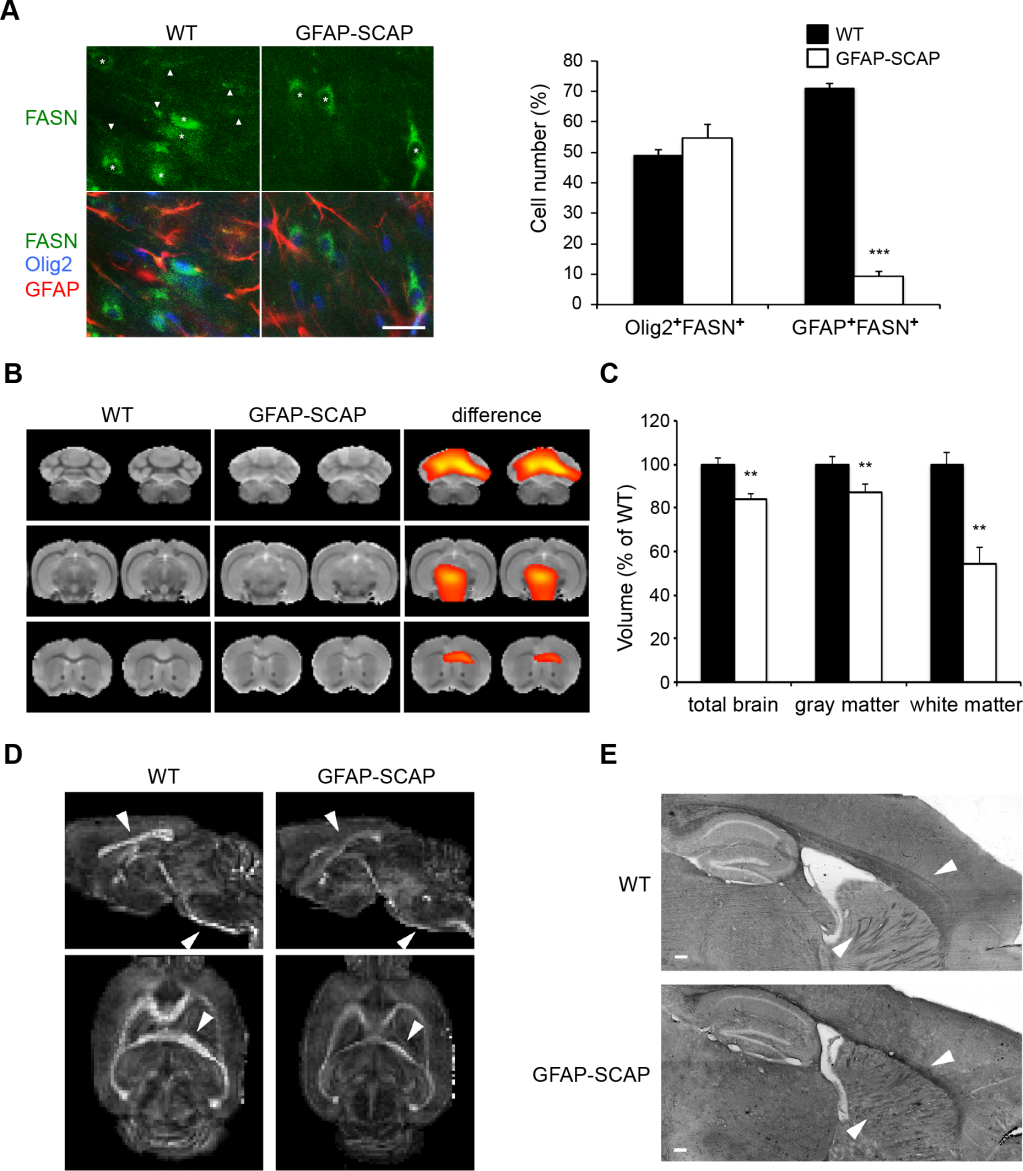
Conditional inactivation of SREBP cleavage-activating protein (SCAP) in astrocytes reduces lipogenic gene expression and white matter volume. **A)** Expression of fatty acid synthase (FASN, green) in oligodendrocytes (Olig2, blue) and astrocytes (glial fibrillary acidic protein [GFAP], red) of wild-type (WT) or GFAP-SCAP mutant mice at P14. Asterisks denote oligodendrocytes with FASN expression; arrowheads denote astrocytes with FASN expression (scale bar, 25 μm). Bar graph shows the number of FASN-positive oligodendrocytes (Olig2^+^FASN^+^) and FASN-positive astrocytes (GFAP^+^FASN^+^) in WT and GFAP-SCAP mice. The values are provided as the percentage of the total number of oligodendrocytes (Olig2^+^ cells) or astrocytes (GFAP^+^ cells). Data are presented as mean ± SEM. ** = *p* < 0.01 using *t* test, *n* = 3. **B)** The average T_2_-weighted magnetic resonance image (MRI), in coronal slices, shows clear diminished white matter in the GFAP-SCAP mice (B, middle) as compared to the average WT image (B, left). Deformation-based morphometry analysis revealed regional differences in tissue volumes in the brains of GFAP-SCAP, mostly in the white matter areas (significant higher volumes in WT shown from red to yellow *p* < 0.05 to 0.0001, *n* = 4–5), overlaid on the average WT image (B, right). See S2 Fig for complete scans. **C)** Volumes of the whole brain and the white and grey matter of GFAP-SCAP and WT mice, as determined with MRI. Volumes are normalized to WT levels that were set to 100%. Data are shown as mean ± SEM (*t* test ** = *p* < 0.01, *n* = 4–5. **D)** Diffusion tensor imaging (DTI) of the same brains as in A, showing reduced fractional anisotropy in GFAP-SCAP mutant brains. Arrowheads show examples of the most affected white matter regions, e.g., corpus callosum, internal capsule, and corticospinal tract. **E)** Sudan Black staining shows less white matter in GFAP-SCAP compared to WT animals (8 months). Arrowheads show the most affected regions, i.e., corpus callosum and internal capsule (scale bar, 200 μm). The numeric data underlying Fig 4A and C can be found in S1 Data.

To determine the role of astrocyte-derived extracellular lipids in myelination, we analyzed glial fibrillary acidic protein (GFAP)-SCAP mice, in which SCAP was deleted from the majority of astrocytes by Cre recombination [11]. Accordingly, the number of astrocytes with FASN expression was strongly reduced in GFAP-SCAP mutants, whereas the number of FASN-expressing oligodendrocytes was not changed (Fig 4A). We previously noticed microcephaly in GFAP-SCAP mice [10]. Structural magnetic resonance imaging (MRI) revealed a large decrease in white matter volume of GFAP-SCAP mutants (to less than 60% of the wild-type [WT] volume), whereas grey matter volume was only reduced by 10% (Fig 4B, 4C and S2A Fig). MRI-based 3D reconstructions of GFAP-SCAP mutant brains showed the most pronounced reduction in the corpus callosum (S2B Fig). Using diffusion tensor imaging (DTI) we found a lower degree of fractional anisotropy for the main tracts in GFAP-SCAP mutant brains compared to WT (Fig 4D). Reduced fractional anisotropy, a measure for axon fiber bundle packing [26,27], in GFAP-SCAP mutants likely reflects a reduction in the number of myelin tracts. In line with this, Sudan Black staining of lipid-rich structures showed smaller white matter structures, particularly in the corpus callosum and internal capsule (Fig 4E). No changes in hippocampal or cortical region sizes were observed, in line with previous observations [11]. Taken together, SCAP deletion in astrocytes leads to reduced and less well-structured CNS white matter tracts.

**Fig 5:**
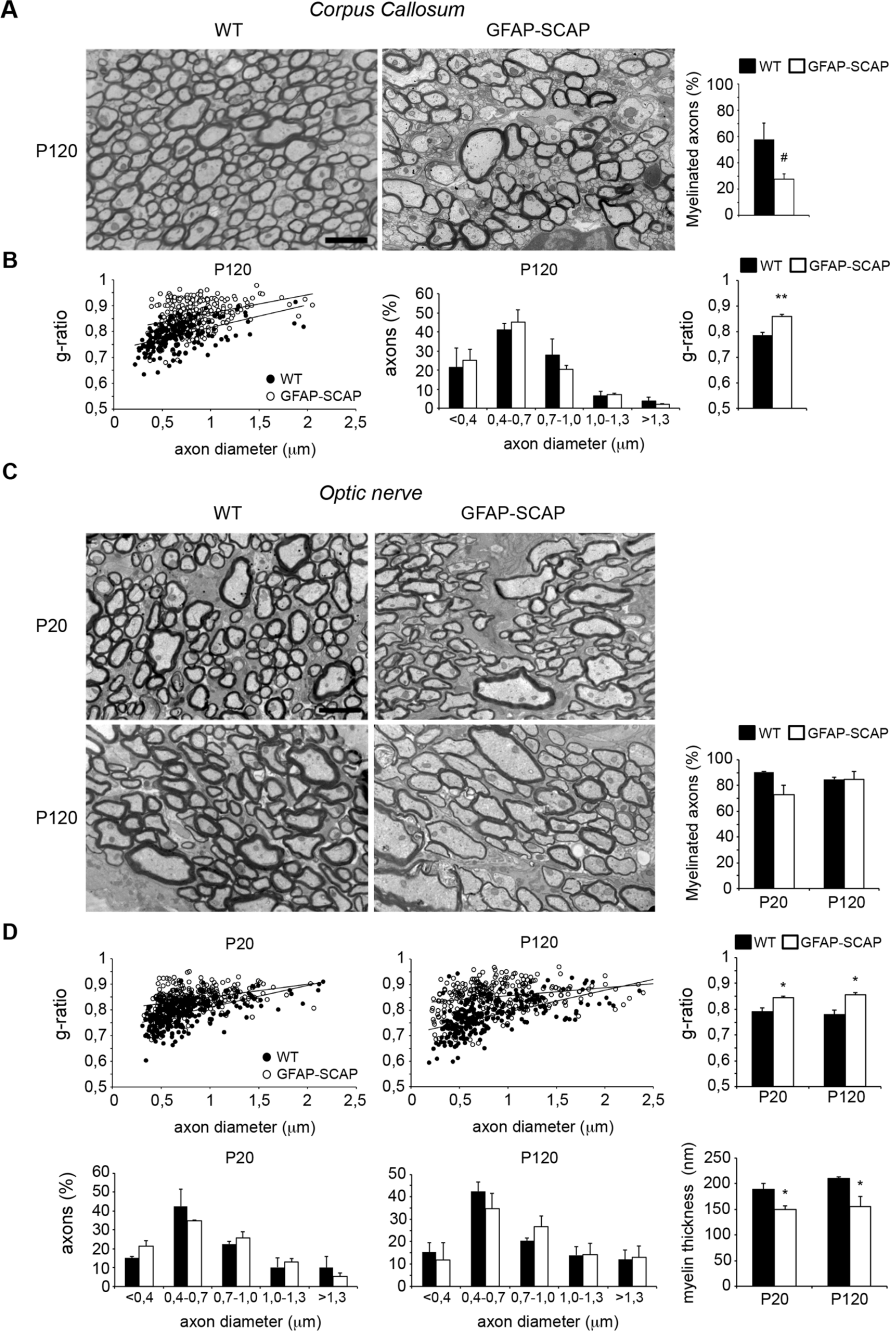
Persistent hypomyelination in glial fibrillary acidic protein (GFAP)- SREBP cleavage-activating protein (SCAP) mutant brains. **A)** Electron microscopy (EM) analysis of corpus callosum myelination in cross-sections of either wild-type (WT) or GFAP-SCAP mice at P120. Bar graph shows the percentage of axons that is myelinated. **B)** Morphometric analysis of axons on corpus callosum of WT and GFAP-SCAP mice, showing g-ratio (myelinated axons) and axonal size distribution (both myelinated and non-myelinated axons) at P120. The relation between axon diameter (x) and g-ratio (y) was y = 9E − 05x + 0.7287 for WT and y = 7E − 05x + 0.8008 for GFAP-SCAP, with coefficients of determination R^2^ = 0.25384 (WT) and 0.11285 (GFAP-SCAP). **C)** EM analysis of optic nerve myelination in cross-sections of either WT or GFAP-SCAP mice at depicted time points. Bar graph shows the percentage of axons that is myelinated. **D)** Morphometric analysis of axons on optic nerves of WT and GFAP-SCAP mice, showing g-ratio (myelinated axons), axonal size distribution (both myelinated and nonmyelinated axons), and myelin membrane thickness at P20 and P120. At P20, the relation between axon diameter (x) and g-ratio (y) was y = 8E − 05x + 0.7262 for WT and y = 5E − 05x + 0.8003 for GFAP-SCAP, with coefficients of determination R^2^ = 0.31108 (WT) and 0.11441 (GFAP-SCAP). At P120: y = 9E − 05x + 0.7079 (WT); y = 3E − 05x + 0.825 (GFAP-SCAP), R^2^ = 0.3288 (WT) and R^2^ = 0.06062 (GFAP-SCAP). Scale bars, 2 μm. *t* test # *p* = 0.079, * *p* < 0.05, ** *p* < 0.01, *n* = 3. The numeric data can be found in S1 Data.

Further analysis revealed a reduced density of corpus callosum myelinated fibers in adult GFAP-SCAP mutants relative to control animals (Fig 5A) due to the absence of myelin around the small diameter axons (<0.5 μm). Moreover, myelin of the large diameter callosum axons was thinner, as demonstrated by a higher g-ratio in GFAP-SCAP mutants. No changes in axonal diameter were found (Fig 5B). Analysis of the optic nerves showed that GFAP-SCAP nerves were also hypomyelinated, although the percentage of myelinated axons was not significantly affected (Fig 5C). G-ratio measurements confirmed that hypomyelination of the optic nerve, particularly for the small diameter axons, was present at P20 and persisted in adults (Fig 5D), whereas no changes in axonal diameter were found.

**Fig 6:**
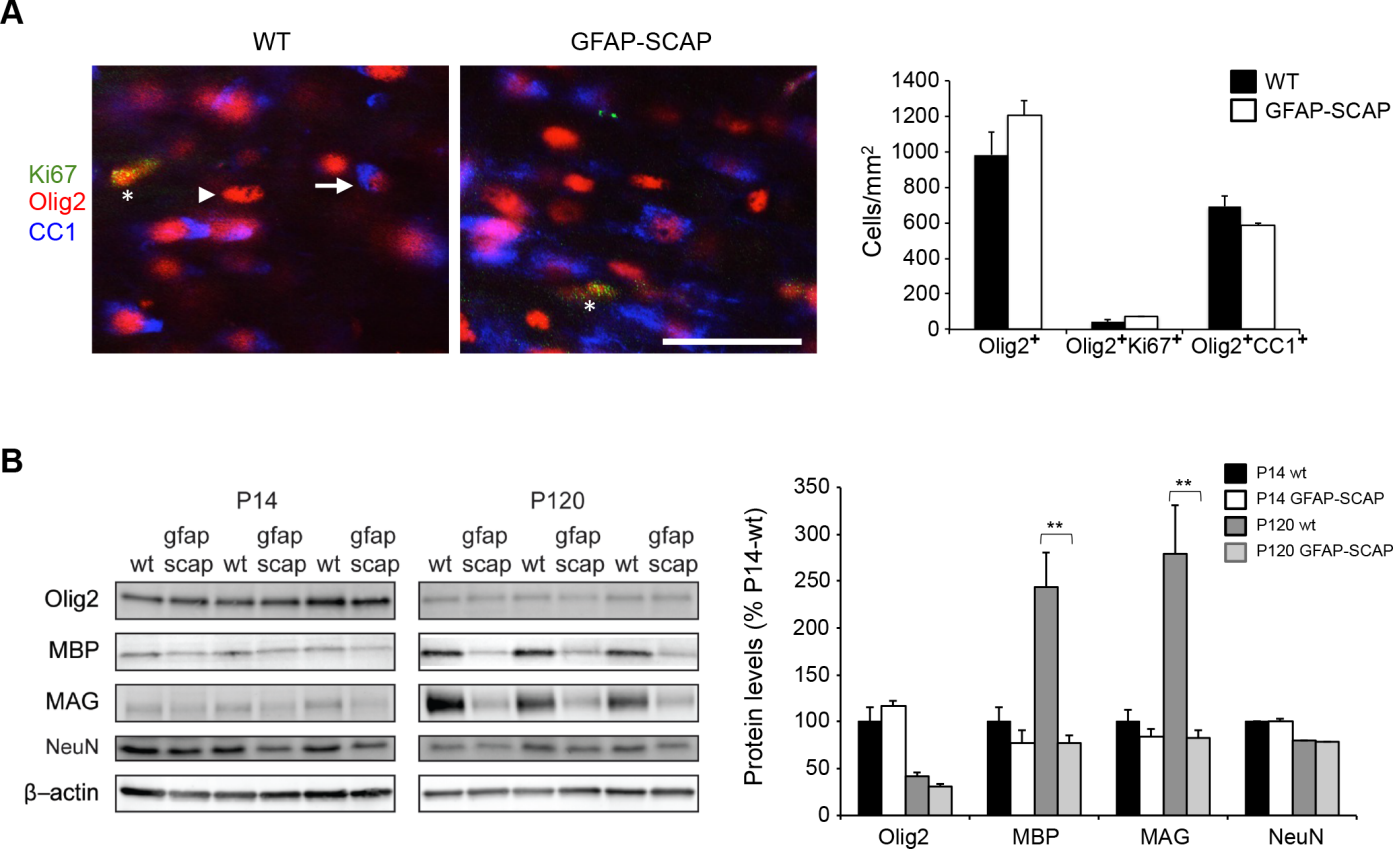
Reduced myelin proteins levels in glial fibrillary acidic protein (GFAP)- SREBP cleavage-activating protein (SCAP) mice. **A)** Expression of postmitotic marker CC1 (blue) or proliferation marker Ki67 (green) in oligodendrocytes (Olig2, red) of wild type (WT) or GFAP-SCAP mutant mice in the corpus callosum at P14. The arrow and arrowhead denotes examples of oligodendrocytes with or without CC1 expression, respectively. The asterisks denote an oligodendrocyte with Ki67 expression (scale bar, 40 μm). The bar graph shows the density of the total number of oligodendrocytes (Olig2^+^ cells), immature oligodendrocytes (Olig2^+^Ki67^+^ cells) and postmitotic mature oligodendrocytes (Olig2^+^CC1^+^ cells) in WT and GFAP-SCAP mice. **B)** Protein levels of Olig2 (oligodendrocyte marker), myelin basic protein (MBP) and myelin-associated glycoprotein (MAG), NeuN (neuronal marker), and β-actin (loading control) were determined by immunoblotting of total brain extracts of WT and GFAP-SCAP mutant animals at P14 and P120. The bar graph shows quantification of protein levels that were first corrected for equal loading using coomassie staining, subsequently normalized to WT levels at P14 and then set to 100%. Data are presented as mean ± SEM. * = *p* < 0.05 ** = *p* < 0.01 using *t* test, *n* = 3. The numeric data can be found in S1 Data.

We previously showed that GFAP-SCAP mice have no changes in neuronal or astrocyte densities [10]. Quantification of Olig2^+^ cell numbers (P14; Fig 6A) showed that GFAP-SCAP mice had no significant changes in the number of OPC/oligodendrocyte cells, CC1^+^Olig2^+^ mature oligodendrocytes, nor in proliferating oligodendrocyte lineage cells (Ki67^+^Olig2^+^ cells) (Fig 6A). The levels of myelin proteins, such as myelin basic protein (MBP) and myelin-associated glycoprotein (MAG), were also reduced in GFAP-SCAP mice at P120 (Fig 6B), whereas smaller reductions in myelin protein levels were found at P14. No changes between WTs and GFAP-SCAP mutants were found for Olig2 and NeuN (Fig 6B). These data demonstrate that astrocyte SCAP mutants have lower numbers of fully myelinating oligodendrocytes.

**Fig 7:**
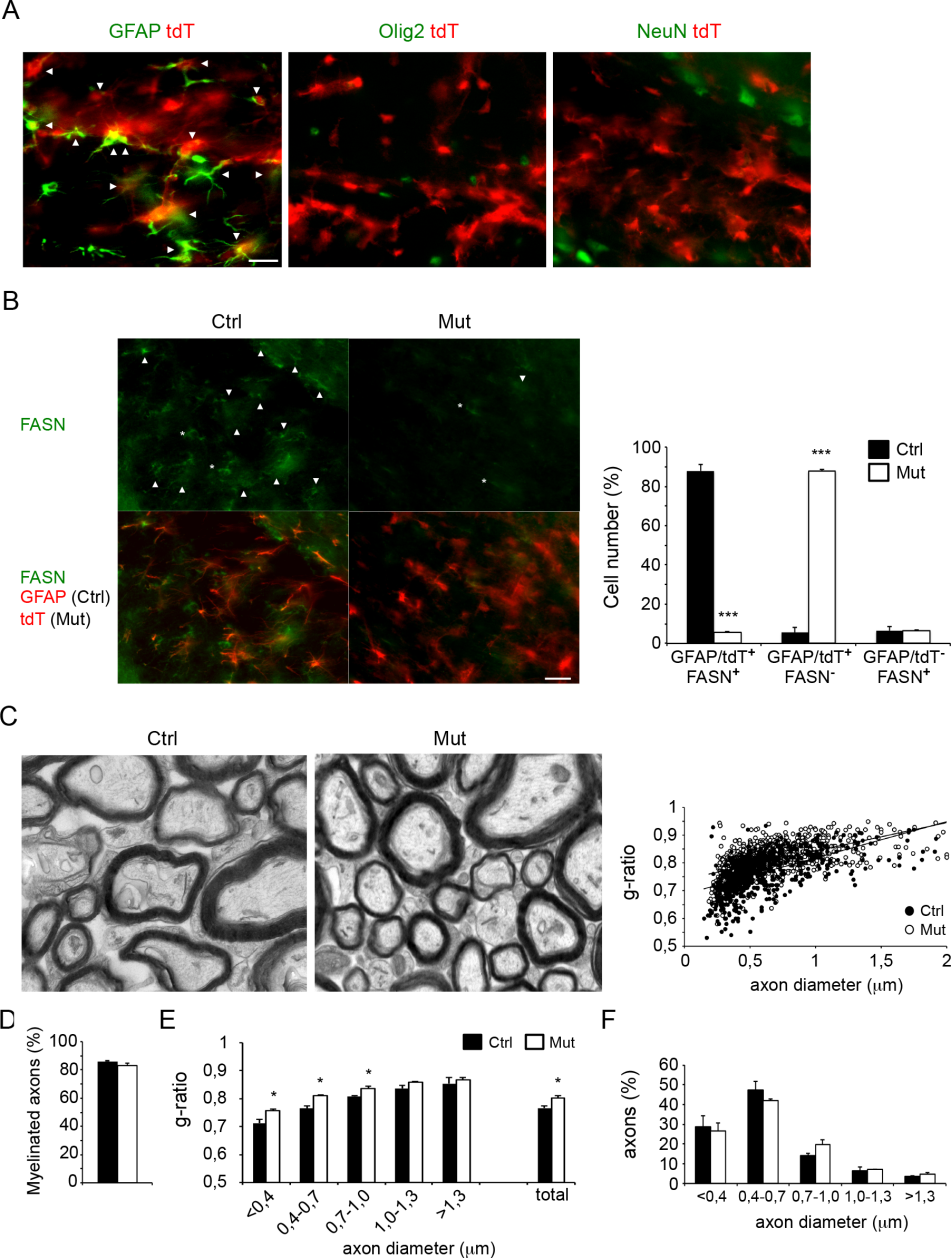
Postnatal tamoxifen-induced inactivation of SREBP cleavage-activating protein (SCAP) in astrocytes interferes with full myelin membrane synthesis. **A)** Expression analysis of recombination-reporter protein td-Tomato (tdT, red) in astrocytes (glial fibrillary acidic protein [GFAP], green), oligodendrocytes (Olig2, green), or neurons (NeuN, green) in the corpus callosum of Glast-CreERT2-tdT-SCAP animals (P56) treated with tamoxifen at P15–P17. Arrowheads denote cells that show coexpression of tdT with cell-type specific markers (scale bar, 25 μm). **B)** Expression of fatty acid synthase (FASN, in green) in astrocytes in tamoxifen-treated control (ctrl, with GFAP in red) or SCAP mutant mice (Mut). Astrocytes are visible by GFAP staining (red, for ctrl mice) or tdT expression (red, Mut). Arrowheads denote FASN+ astrocytes, asterisks denote FASN+ cells that are negative for GFAP or tdT (scale bar, 25 μm). Bar graph shows the number of FASN-positive astrocytes in WT (GFAP^+^FASN^+^ cells) and GFAP-SCAP mice (tdT^+^FASN^+^ cells) or FASN-positive non-astrocyte cells (GFAP^-^FASN^+^ or tdT^-^FASN^+^ cells). The values are provided as a percentage of the total number of cells expressing GFAP, tdT, or FASN. Data are presented as mean ± SEM. *** = *p* < 0.001 using *t* test, *n* = 3. **C)** Electron microscopy (EM) analysis of corpus callosum myelination in cross-sections of either tamoxifen-treated control (ctrl) or SCAP mutant mice (Mut) at P56. The relation between axon diameter (x) and g-ratio (y) was y = 0.1308x + 0.6872 for Ctrl and y = 0.1022x + 0.7408 for Mut, with coefficients of determination R^2^ = 0.32986 (Ctrl) and R^2^ = 0.29418 (Mut). **D)** Bar graph shows the percentage of axons that are myelinated. **E)** Morphometric analysis of axons on optic nerves of Ctrl and SCAP mutant mice showing g-ratio per class of axonal diameter for myelinated axons, and **F)** axonal size distribution for both myelinated and nonmyelinated axons. *t* test * = *p* < 0.05. ** = *p* < 0.01, *n* = 3. The numeric data underlying Fig 7B and C can be found in S1 Data.

To establish a role of astrocytes during a later stage of myelination, we induced SCAP deletion specifically in astrocytes around the developmental peak of myelination (P20) [28]. To accomplish this, Glast-CreERT2-tdT-SCAP mice were injected with tamoxifen at P15–P17, which prevents potential neural progenitor perinatal and early postnatal targeting [29,30]. Glast-CreERT2-tdT mice (P56) had td-Tomato (tdT) reporter gene expression in the corpus callosum in the large majority of GFAP^+^ astrocytes, while virtually no expression was found in Olig2^+^ oligodendrocytes or axons (Fig 7A). Accordingly, FASN expression was strongly reduced in astrocytes of Glast-CreERT2-tdT-SCAP adult mice (Fig 7B). EM showed that the corpus callosum of Glast-CreERT2-tdT-SCAP mutant mice was hypomyelinated at P56 (Fig 7C), without affecting the percentage of myelinated axons (Fig 7D), which predominantly affected the small caliber fibers (Fig 7E). In contrast, axonal diameter was not affected (Fig 7F).

Taken together, these results demonstrate that compromised astrocyte lipid metabolism, also when induced during postnatal development, limits myelin membrane synthesis causing persistent CNS hypomyelination.

### Oligodendrocytes show compensatory incorporation of dietary lipids in the myelin membrane when astrocyte lipid synthesis is compromised

**Fig 8:**
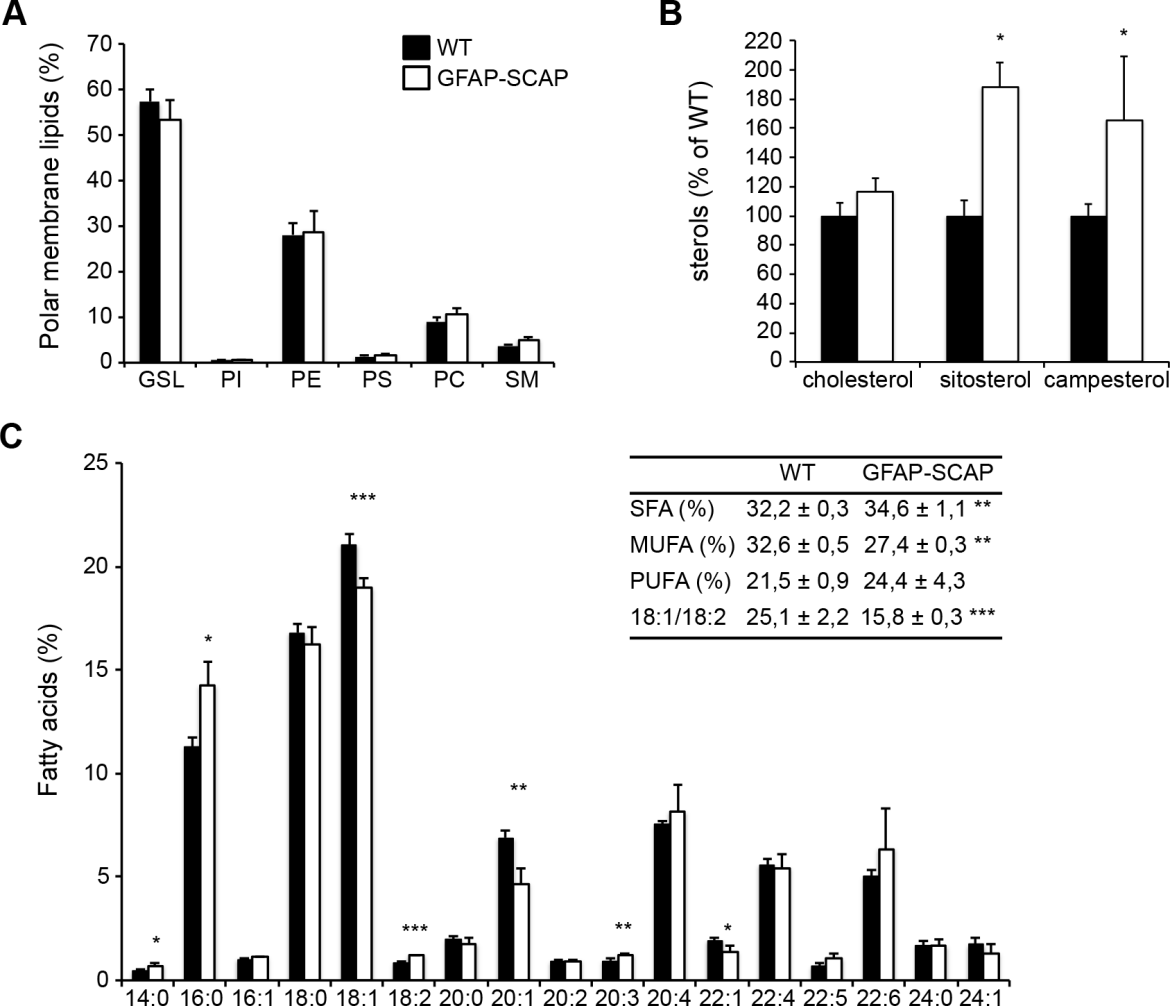
Myelin from glial fibrillary acidic protein (GFAP)-SREBP cleavage-activating protein (SCAP) brains shows increased accumulation of dietary lipids. Lipid extracts of purified myelin of GFAP-SCAP brains (P42) were analyzed using liquid chromatography and mass spectrometry. **A)** Polar membrane lipid concentration and **(B)** sterol concentration per protein amount in GFAP-SCAP compared to wild-type (WT) myelin. GSL, glycosphingolipid; PI, phosphatidyl inositol; PE, phosphatidyl ethanolamine; PS, phosphatidyl serine; PC, phosphatidyl choline; SM, sphingomyelin. **C)** Fatty acid profile of phospholipids from purified myelin with the amount of different fatty acids species as the percentage of the total amount. Fatty acid species are depicted as “y:z,” with “y” representing the length of the fatty acid molecules and “z” representing the number of double bonds. Insert: depicted are proportions of saturated fatty acids (SFA), monounsaturated fatty acids (MUFA), polyunsaturated fatty acids (PUFA), and the ratio of 18:1/18:2. Data are presented as the mean percentage of WT ± SD. *t* tests: * = *p* < 0.05; ** = *p* < 0.01; *** = *p* < 0.001, *n* = 4. The numeric data can be found in S1 Data.

Lipid analysis of purified myelin from GFAP-SCAP brains (P42) revealed no changes in phospholipid classes (Fig 8A) or cholesterol (Fig 8B), which was similar to our observations in CNP-SCAP mutant mice (cf. Fig 3). Interestingly, however, GFAP-SCAP myelin membranes contained more sitosterol and campesterol (Fig 8B), albeit at trace levels compared to cholesterol (in mutant myelin: 0.66 and 2.64 pmol/ug protein of resp. sitosterol and campesterol versus 2.07 nmol/ug protein cholesterol). Since sitosterol and campesterol are 2 plant sterols that can only be derived from diet, this finding suggested that GFAP-SCAP mutants unexpectedly incorporated plasma-derived sterols into myelin. The fatty acid composition of phospholipids was significantly shifted from monounsaturated fatty acids towards polyunsaturated fatty acids, and a decrease in the ratio of 18:1/18:2 in mutant myelin was observed (Fig 8C). As observed in CNP-SCAP mice (cf. Fig 3), GFAP-SCAP mutant myelin also contained more polyunsaturated fatty acids and had higher levels of the essential fatty acid C18:2, which is consistent with an increased uptake of fatty acids from external sources[5,6]. In this manner, compromised lipid metabolism in astrocytes leads to a reduction in myelin membrane synthesis, as well as a compensatory increase in oligodendrocyte uptake of sterols and fatty acids from the circulation.

**Fig 9:**
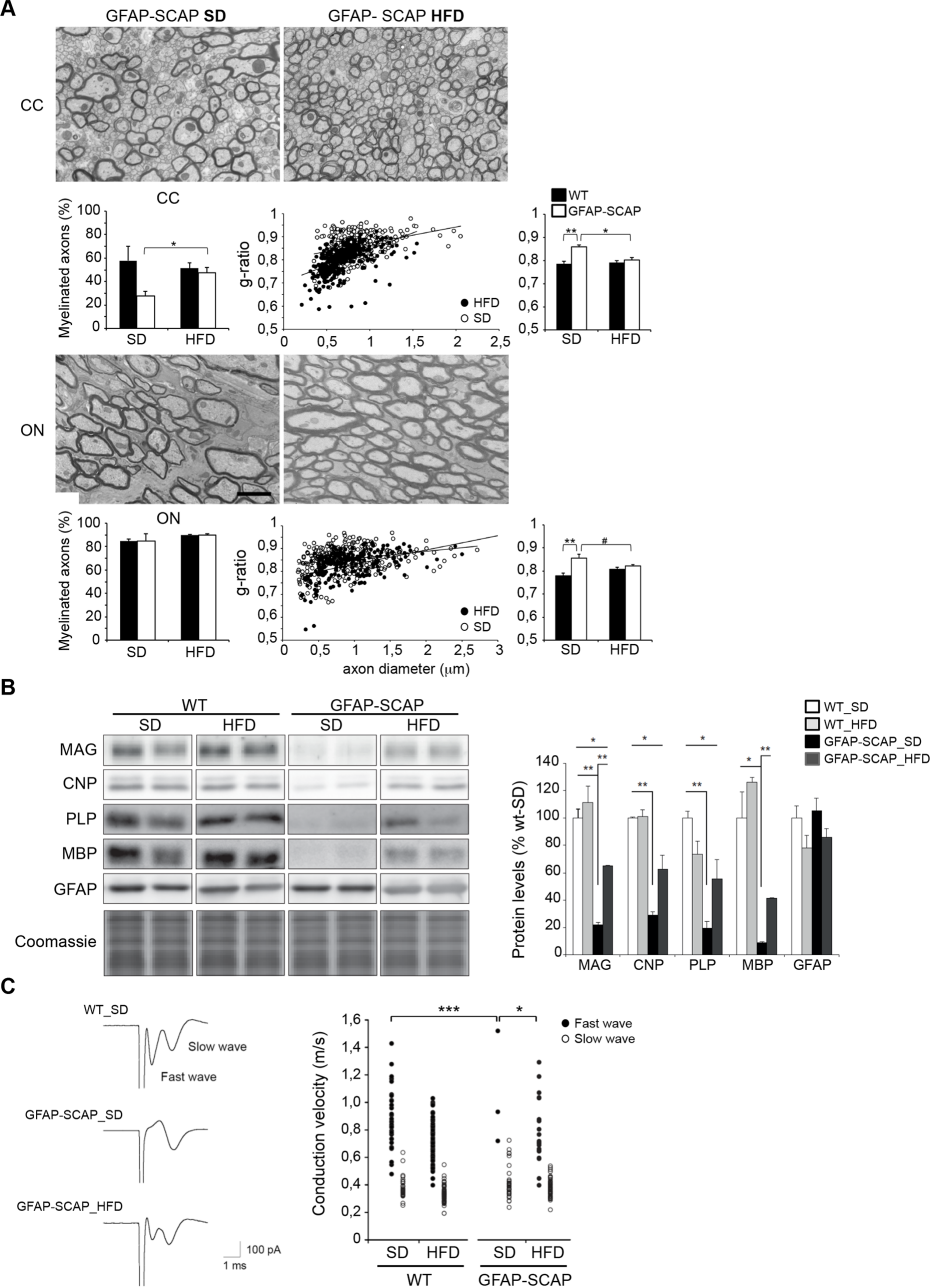
Effects of a high-fat diet (HFD) on myelination, myelin protein levels, and white matter conduction velocity. **A)** Electron microscopy (EM) analysis of optic nerve (ON) and corpus callosum (CC) myelination in cross-sections of glial fibrillary acidic protein (GFAP)-SREBP cleavage-activating protein (SCAP) mice (P120) on either a standard diet (SD) or HFD. See Fig 5A and 5C for representative EM images of wild-type (WT) animals (P120) on SD or HFD. Scale bar, 2 μm. Bar graphs, percentage of axons that are myelinated (left). Morphometric analysis of myelinated axons showing g-ratio (middle and right). For ON of GFAP-SCAP mice, the relation between axon diameter (x) and g-ratio (y) was y = 3E − 05x + 0.825 for SD and y = 6E − 05x + 0.7687 for HFD, with coefficients of determination R^2^ = 0.06062 (SD) and R^2^ = 0.23735 (HFD). For CC of GFAP-SCAP mice, the relation between axon diameter (x) and g-ratio (y) was y = 7E − 05x + 0.8008 for SD and y = 0.0001x + 0.701 for HFD, with coefficients of determination R^2^ = 0.11285 (SD) and R^2^ = 0.26559 (HFD). *t* test, ** *p* < 0.01, * *p* < 0.05, # *p* = 0.07, *n* = 3–4. **B)** Immunoblot against depicted myelin proteins and coomassie staining of protein levels of total brain extracts of GFAP-SCAP mutant and WT mice (P120) fed with SD or HFD. Right panel: quantification of immunoblot for depicted myelin proteins for GFAP-SCAP and WT mice fed with SD or HFD (*n* = 3). Coomassie staining was used for normalization. Data are presented as mean ± SEM, in which WT-SD levels were set to 100%. *t* test * *p* < 0.05; ** *p* < 0.01). **C)** Example of compound action potential waveforms in the CC for a WT mouse on a standard diet (WT-SD), and a GFAP-SCAP mutant mouse on a standard diet (GFAP-SCAP-SD) or high fat diet (GFAP-SCAP-HFD). Right panel: individual plots of conduction velocity measurements in the CC of GFAP-SCAP mutant and WT fed with SD or HFD. Chi-square test, *n* = 12–17, * *p* < 0.05, *** < 0.001. The numeric data can be found in S1 Data.

Next, we tested whether hypomyelination in these mice could be rescued by further increasing dietary lipid intake. We previously showed that GFAP-SCAP mice treated from E15 onwards with a high-fat diet (HFD), enriched in cholesterol and fatty acids, improved motor deficits and survival of the mutant mice [11]. Here we show that HFD treatment rescued hypomyelination, as shown for the optic nerve and the corpus callosum at P120 (Fig 9A), and increased the levels of myelin proteins, e.g., MAG, myelin proteolipid protein (PLP), CNP, and MBP in brains of GFAP-SCAP mutants, while GFAP protein levels were not changed (Fig 9B). To determine whether HFD treatment also led to functional recovery of myelin tracts, we measured action potential conduction velocity (CV) in the corpus callosum. In the majority of corpus callosum slices from WT animals tested, extracellular stimulation evoked compound action potentials that showed 2 discrete propagation speeds (0.86 ± 0.03 m/s “fast wave,” *n* = 36, and 0.38 ± 0.02 m/s “slow wave,” *n* = 27; Fig 9C). These were most likely generated by myelinated axons (fast wave) and nonmyelinated axons (slow wave), respectively. In GFAP-SCAP animals, the fast wave was absent in more than 95% of the corpus callosum slices (*p* < 0.001, chi-square test), while the slow wave was unaffected (Fig 9C). Thus, SCAP deletion in astrocytes most likely specifically affected action potential propagation in myelinated axons. Interestingly, treatment with HFD increased the number of fast responses in GFAP-SCAP animals from 5% (in standard diet) to 50% (*p* = 0.03), whereas no effect on conduction velocity was observed in WT animals (0.79 ± 0.05 m/s, *n* = 19, *p* > 0.05). Thus, a HFD partially rescues both myelination and conduction velocity of GFAP-SCAP mutants. Altogether, our results show that compromised lipid metabolism in astrocytes leads to CNS hypomyelination, which can be overcome structurally and functionally by a high-fat diet.

### Myelination is virtually blocked when lipid synthesis is compromised in both oligodendrocytes and astrocytes

**Fig 10:**
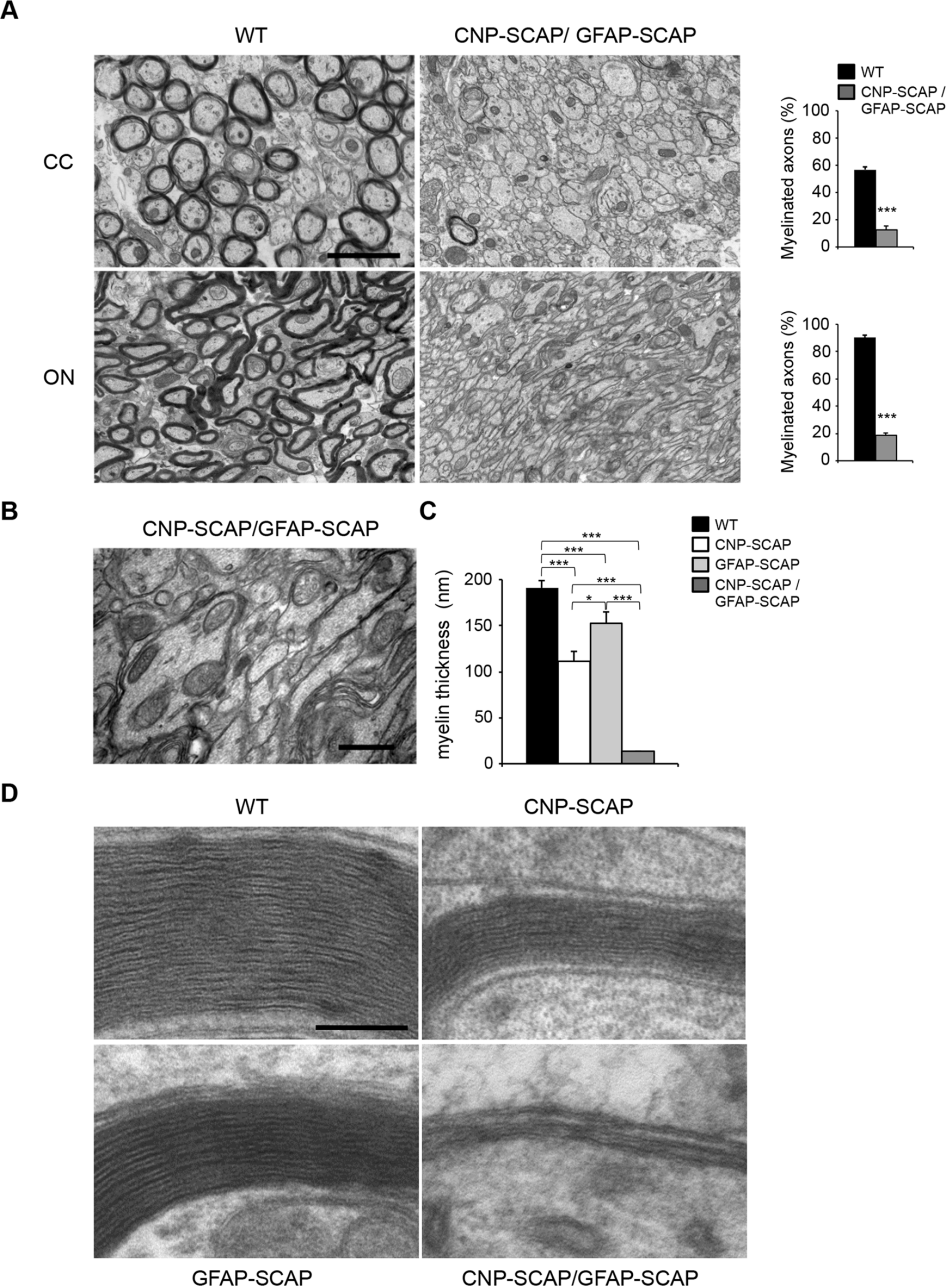
Virtually no myelin membrane synthesis in CNP-SREBP cleavage-activating protein (SCAP)/glial fibrillary acidic protein (GFAP)-SCAP brains. **A)** Electron microscopy (EM) analysis of the corpus callosum (CC) and optic nerve (ON) in P20-old wild-type (WT) mice or mice carrying a deletion in both astrocytes and oligodendrocytes (CNP-SCAP/GFAP-SCAP). Bar graphs show the percentage of axons that are myelinated. **B)** Enlarged view on part of the electron micrograph of CNP-SCAP/GFAP-SCAP ON in A. **C)** morphometric analysis of myelinated axons in the ON of WT, CNP-SCAP, GFAP-SCAP, and CNP-SCAP/GFAP-SCAP mice at p20, showing myelin membrane thickness. *n* = at least 3 animals. Membrane thickness for each CNP-SCAP/GFAP-SCAP animal was determined for at least 22 axons that were wrapped by oligodendrocyte membrane, as depicted in 10B and D. **D)** Electron microscopic analysis of myelin membranes in the ON of WT, CNP-SCAP, GFAP-SCAP, and CNP-SCAP/GFAP-SCAP mice. Data are presented as mean ± SEM. *t* test * *p* < 0.05, *** *p* < 0.001, *n* ≥ 3. Scale bar, (A) 2 μm, (B) 0.75 μm, (C) 0.1 μm. The numeric data underlying Fig 10A and C can be found in S1 Data.

Our observations imply that CNS myelin membrane synthesis not only requires endogenous oligodendrocyte lipid synthesis but also crucially depends on extracellular lipids provided by astrocytes. We therefore created CNP-SCAP/GFAP-SCAP animals, in which SCAP was deleted in both oligodendrocytes and astrocytes. CNP-SCAP/GFAP-SCAP mutant mice were born at expected ratios and could phenotypically not be distinguished from WTs. However, animals soon developed motor deficits and reduced weight gain more severe than single CNP-SCAP mice, and all mice died or reached a humane endpoint requiring euthanasia between P15–P21. The corpus callosum and optic nerves of CNP-SCAP/GFAP-SCAP animals (P20) were practically devoid of myelin (Fig 10A). Oligodendrocytes ensheathed large caliber fibers, but failed to make more than a few membrane layers (Fig 10B). Accordingly, whereas myelin membranes were thinner in CNP-SCAP and GFAP-SCAP animals, they were nearly absent in CNP-SCAP/GFAP-SCAP animals (Fig 10C and 10D). Treatment of dams with HFD did slightly increase the body weight of their CNP-SCAP/GFAP-SCAP pups. Nevertheless, all animals died or reached a humane endpoint between P15–P21, and analysis of white matter showed no improvement in hypomyelination (S3 Fig), suggesting that the resulting developmental defect was too severe to be rescued by dietary lipid supplementation. Taken together, myelin membrane synthesis shows different kinetics when lipid synthesis is compromised in either oligodendrocytes or astrocytes, whereas it is virtually absent when lipid synthesis is compromised in both cell types.

## Discussion

We showed that SCAP is required for the activation of SREBP-mediated lipogenic gene expression in oligodendrocytes and observed that oligodendrocyte SCAP mutant brains are hypomyelinated during the regular peak of myelination and slowly regain close-to-normal levels of myelin membrane with age. These data are in line with previous observations in CNPcre-SQS mice, in which cholesterol-deficient oligodendrocytes are able to slowly synthesize myelin[7,31], although recent observations indicate that targeting of neuronal populations in the cortex cannot be excluded[32]. Our data reveal that under conditions of compromised oligodendrocyte lipid synthesis, the extracellular lipids are supplied by astrocytes. Furthermore, our data reveal that full myelin membrane synthesis requires an astrocyte lipid supply in addition to endogenous oligodendrocyte lipid synthesis. Moreover, when astroglial lipid synthesis was selectively compromised, oligodendrocytes incorporated circulating lipids into the myelin membrane, and a lipid-enriched diet rescued hypomyelination, showing lipid flux from the circulation to the myelin membrane under astrocyte metabolism-compromised conditions.

### Involvement of astrocyte lipid metabolism in myelin membrane synthesis

We previously reported that GFAP-SCAP mutant mice have microcephaly without changes in neuronal and astrocyte density[11]. Here, we show by structural MRI and DTI that volume reduction was mostly pronounced in the white matter. Cells of the oligodendrocyte lineage were not reduced in density; instead, the formation of fully developed myelin membranes was lower in the GFAP-SCAP mutant, resulting in a functional loss of fast conduction myelinated fibers. We conclude that GFAP-SCAP mutant mice have white matter atrophy that is caused by persistent hypomyelination.

Although GFAP-SCAP mice showed clear CNS hypomyelination in adults (P120), hypomyelination was less pronounced in younger mutant animals. This suggests that depletion of astrocyte lipids becomes most limiting after the first phase of myelination. Indeed, astrocyte-specific deletion of SCAP, using Glast-CreERT2-tdT-SCAP mice, late in this developmental phase of myelination prevents the establishment of a full myelin membrane in adults. In line with this observation, oligodendrocytes produce large amounts of cholesterol during the peak of myelination, but, thereafter, cholesterol synthesis occurs mainly in astrocytes[33,34]. It should be noted that the myelin membrane surface increases exponentially with increasing fiber diameter, during both myelin membrane wrapping and developmental axonal radial growth[35], which may underlie the elevated need for astrocyte lipids at a later stage of myelination. A role for astrocytes in the later stages of myelination is not unprecedented, as it was previously reported that astrocytes support myelin membrane synthesis in vitro, as opposed to OPC differentiation or initial myelin membrane wrapping[19]. Interestingly, astrocytes are in contact with axons at the node and promote myelination in response to electrical impulses [36]. This finding indicates a role for astrocytes in activity-dependent myelination, a process that may underlie myelin plasticity relevant to learning in adults [3,36]. Whether the supply of lipids from astrocytes to oligodendrocytes is regulated by axonal activity and is involved in activity-dependent myelination in adults remains to be determined.

Our observation that hypomyelination in GFAP-SCAP mutants is more pronounced for small-diameter axons might be related to the finding that large axons are the first to be myelinated during development [37]. Therefore, under conditions in which lipid supply is limited, e.g., when astrocyte-derived lipid supply is compromised, oligodendrocytes that enwrap large axons are in favor to use the small amount of lipids initially available. The virtual absence of myelin around each axon when SCAP is deleted in both oligodendrocytes and astrocytes shows that these 2 cell types are the main lipid contributors for the oligodendrocyte myelin membrane.

### Implications for understanding and dietary treatment of white matter diseases

We propose that endogenous lipid levels in oligodendrocytes are sufficient for initial myelin membrane synthesis in the first postnatal weeks, while subsequent elaboration of a full myelin membrane requires lipid supply from astrocytes. Importantly, feeding astrocyte-lipid mutants with a cholesterol- and oleic acid-enriched diet led to an increase in myelination; in particular, small-diameter axons did benefit from this treatment. This indicates that lipids, with elevated circulation levels, can reach the brain and are incorporated in the growing myelin membrane, as we found for dietary sterols and essential fatty acids. The inability of a lipid-enriched diet to improve myelination in CNP-SCAP/GFAP-SCAP mice may be related to the severity of the developmental defect or their life span being too short for the diet to be effective. The exact mechanisms by which this diet improved myelination in GFAP-SCAP mice remains to be determined but may involve the close vicinity of astrocytes end-feet to blood capillaries and thereby the uptake of circulating lipids by astrocytes and subsequent delivery of lipids to oligodendrocytes. It should be noted that although horizontal cholesterol transfer was suggested to improve myelination in CNP-SQS mutant mice, a cholesterol-enriched diet did not improve myelination in these mice [7], probably because oligodendrocytes do not have the same access to circulating lipids as astrocytes. As such, we observed that dietary sterols (phytosterols) were incorporated in GFAP-SCAP mutant myelin but not in CNP-SCAP mutant myelin. Our results indicate that the extent of exogenous lipid uptake by oligodendrocytes for myelin membrane synthesis has been underestimated. Without astrocyte lipid synthesis, oligodendrocytes are unable to finalize CNS myelination, leading to hypomyelinated and slower-conducting fibers in adulthood. These data may have important implications in the understanding and treatment of myelin diseases. Some of the myelin defective phenotypes are known to benefit from dietary supplemented lipids, (SLOS, X-linked Adrenoleukodystrophy), however, with mixed effects in different patients, which calls for optimization and detailed understanding of the underlying mechanisms[38,39]. Considering the need of lipids for myelination and remyelination, our findings show that oligodendrocytes depend on astrocyte lipid metabolism, or on lipids supplemented in the diet under astrocyte metabolism-compromised conditions, which might be instrumental for the development of novel strategies aimed at restoring loss of function in myelin diseases.

## Methods

### Mice

All experimental procedures were approved by the local animal research committee (Dierexperimentencommissie VU University, protocols: MCN10-04, MCN10-20, MCN12-16, MCN13-01; MCN14-16) and complied with the European Council Directive (86/609/EEC). All animals were housed and bred according to the institutional and Dutch governmental guidelines for animal welfare. Extra care was taken of animals that suffered from genotypic phenotypes and experimental procedures, including the use of humane endpoints.

SCAP-floxed mice were from the Jackson Laboratory and have been described[22]. The hGFAP-Cre-IRES-LacZ transgenic mice, referred to as GFAP-Cre, predominantly targets Cre-mediated recombination in astrocytes[40] and only minor populations of neurons in the hippocampus[41], cortex[41], and cerebellum[42]. CNP-cre mice have been described [25]. Glast-CreERT2 mice [29] and Rosa26-tdTomato mice [43] have been described and maintained by breeding with SCAP loxP mice as Glast-creERT2-tdTomato-SCAP mice. Throughout the text, mice of the GFAP-cre/SCAPloxP/loxP genotype were referred to as “GFAP-SCAP” mice, mice of the CNP-cre/SCAPloxP/loxP genotype as “CNP-SCAP” mice, and mice of the CNP-cre/GFAP-cre/SCAPloxP/loxP genotype as “CNP-SCAP/GFAP-SCAP” mice. CNP-SCAP/GFAP-SCAP mice were obtained by breeding of GFAP-Cre(tg/0)//CNP-Cre(tg/0)//SCAPf/+ mice with GFAP-Cre(0/0)//CNP-Cre0/0)//SCAPf/f mice. Littermates of CNP-SCAP/GFAP-SCAP mice that were not homozygous for either CNP-SCAP, GFAP-SCAP, or both were taken as controls. Mouse lines were maintained on a C57Bl6 background. Unless indicated otherwise, food (Harlan Teklad, Madison, WI, USA) and water were provided ad libitum.

### Tamoxifen treatment

Tamoxifen (Sigma-Aldrich) was dissolved in corn oil to a final concentration of 10 mg/ml. Pups received a total amount of 10 μl per gram body weight through intraperitoneal injections for 3 consecutive days at P15–P17.

### Diets

Pregnant mice, on day 14 of gestation, were randomly separated in 2 groups. Group 1 received the standard diet (Teklad diets, Harlan Laboratories, Madison, WI, USA), group 2 received a high-fat diet containing 60% fat calories (1% cholesterol, 31% lard and 3% soybean oil, TD.09167, Teklad diets). Fatty acid content of the diets has been described[11]. Pregnant mice received the diets from the last week of gestation until weaning (3 weeks after birth). Animals were separated at weaning, housed by gender, and continued to receive the same diet.

### MRI and DTI

One-year-old WTs (*n* = 4) and GFAP-SCAP mutant mice (*n* = 5), were perfused transcardially under deep anesthesia with 20 ml of PBS 0.1 M, pH 7.4 containing 0.1% heparin followed by 100 mL of freshly prepared cold fixative solution composed of 4% paraformaldehyde in 0.1 M PBS, pH 7.4 [11]. Brains were removed, postfixed overnight in fixative solution at 4°C, and cryoprotected with 30% sucrose for 2–3 days at 4°C [11]. Post mortem brains were fixated in a syringe filled with perfluoro polyether (Fomblin, Solvay Solexis) to prevent magnetic susceptibility artifacts at the borders of the brain. High resolution DTI was performed to assess white matter status, using a diffusion-weighted eight-shot spin-echo EPI sequence (TR/TE = 2700/28 ms; field-of-view 20×20 mm; 156×156 μm voxels; 91×150 μm coronal slices; b = 2035.5 s/mm^2^, δ = 5 ms, Δ = 13 ms; 2 sets of 60 diffusion-weighted images in noncollinear directions, and 4 unweighted images [b = 0]). The diffusion tensor for each voxel was calculated based on the eigenvectors and eigenvalues using multivariate fitting and diagonalization. Derived fractional anisotropy (FA) maps were further analyzed basically as previously described[44] using unbiased whole-brain tract-based spatial statistics[45]. Image-based registration was performed with Elastix[46]. FA maps of all animals were first aligned to a common reference image using nonlinear registration of the average diffusion-weighted image with limited degrees of freedom preceded by affine-only registration. The transformations that were obtained from the nonlinear registration describe the local tissue volume changes that are needed to match the images to the common reference. At the voxel level, volume expansion or compression was quantified by the determinant of a transformation’s Jacobian matrix. Local tissue volumes were then tested in a voxel-wise deformation-based morphometry analysis[47]. By thresholding the mean FA maps at 0.2, a skeleton of white matter tracts was obtained shared across subjects. With a perpendicular search algorithm, subject FA maps were registered, starting from the skeleton towards individual tracts, and subsequently stacked into a sparse skeletonized 4D image. Permutation tests with threshold-free cluster enhancement [45] were conducted for each point at the mean FA skeleton to assess statistically significant differences between mutant and control groups.

### Conduction velocity

Mice were decapitated, and the brains were rapidly removed and immersed in ice-cold artificial cerebrospinal fluid (ACSF; containing NaCl 129 mM, KCl 3 mM, MgSO_4_ 1.8 mM, CaCl_2_ 1.6 mM, glucose 10 mM, NaH_2_PO_4_ 1.25 mM, NaHCO_3_ 21 mM; pH 7.4) carboxygenated with 5% CO_2_ and 95% O_2_. Coronal slices (400 μm) were acutely prepared from the frontal cortex, including corpus callosum and hippocampus. After sectioning, slices were maintained at 21°C and recorded at room temperature (20°C–22°C) in a similar solution. Extracellular field currents were recorded with Heka EPC-8 amplifiers (D-67466 Lambrechtt/Pfalz, Germany). The ACSF-filled glass microelectrodes were voltage clamped at 0 mV. The measurements were taken from three different locations along the corpus callosum with platinum/iridium electrodes (FHC, Bowdoin, ME 04287, USA). Data were low-pass filtered at 5 kHz, digitized at 20 kHz, with an instrutech ITC-16 and pulse software (D-67466 Lambrecht/Pfalz, Germany) and analyzed off-line with Igor Pro (Wavemetrics, 10200 SW Nimbus, G-7, Portland, USA). Evoked action currents were measured using 2 different recording electrodes and were both abolished by tetrodotoxine (TTX), a selective blocker of voltage-gated sodium channels (S4 Fig).

### Lipid analysis

Myelin was purified by density gradient centrifugation [48], and lipids were isolated by lipid extraction, as described previously [6]. Analysis of neutral lipids was done using a Sciex 4000 Q-trap mass spectrometer (AB Sciex, Framingham, MA, USA), equipped with an atmospheric pressure chemical ionization source. Analysis of free fatty acids was done after mild alkaline hydrolysis of isolated phospholipid fractions from lipid extracts, as described previously[5,6]. Analysis of intact phospholipids were analyzed using defined molecular species and authentic free fatty acid standards, as described previously[5,6].

### Statistical analysis

Statistical differences were analyzed using Student’s *t* test, unless otherwise indicated in the legends. Statistical numeric data are provided in the legends. Data are presented as mean ± SEM.

### Additional methods

Description of additional methods, including EM, morphometric analysis, immunoblotting, and immunohistochemistry are available in S1 Text.

## Supporting information

S1 Fig A) Expression of fatty acid synthase (FASN, green) in WT mice at P21. CC: Corpus Callosum; CA: Cornu Ammonis; DG: Dentate Gyrus (scale bar, 250 μm). B) Expression of FASN (green) is not localized in neurons (NeuN, red) in the cortex (scale bar, 25 μm). C) Expression of FASN (green) is not localized in neurons (NeuN, red) in the CA1 region of the hippocampus (scale bar, 25 μm).
(TIF)

S2 Fig A) Data from 3D MRI brain scans of 1-year old GFAP-SCAP and WT mice. Deformation-based morphometry analysis revealed regional differences in tissue volumes in the brains of GFAP-SCAP in the cerebullum, thalamus, and anterior corpus callosum. Significant localized higher volumes in WT (n = 4) as compared to GFAP-SCAP (n = 5) are shown as red to yellow voxels (p < 0.05 to 0.0001). Significant lower volumes are shown in blue to light blue voxels (p < 0.05 to 0.0001). The statistical maps are overlaid on the coronal slices of the average T2-weighted wild-type image (from posterior to anterior). B) Voxel-based analysis of fractional anisotropy map is shown in green. Statistical comparison was restricted to a white matter skeleton (white; fractional anisotropy > 0.2) using tract-based spatial statistics. Significant lower fractional anisotropy voxels in the GFAP-SCAP mice as compared to WT mice are shown in blue (p < 0.05; false discovery rate-corrected). These voxels are in particular located in the corpus callosum.
(TIF)

S3 Fig Effect of HFD on myelin membrane synthesis in CNP-SCAP/GFAP-SCAP brains. EM analysis of A) corpus callosum (CC) and B) optic nerve (ON) in P20 WT mice or mice carrying a deletion in both astrocytes and oligodendrocytes (CNP-SCAP/GFAP-SCAP), on standard diet (SD) or high fat diet (HFD). Scale bar, 2 μm.
(TIF)

S4 Fig Experimental design of conduction velocity experiments. A) Shown is the position of the action potential evoking electrode (labelled with "Stim") and the 2 recording electrodes ("Rec 1," and "Rec 2"). B) Example of the recorded signal showing in blue the signal recorded with electrode 1, and in orange the signal recorded with electrode 2. C) Inhibition of the signal by 1 μm tetrodotoxin (TTX) applied in the bath before stimulation.
(TIF)

S1 Text Description of additional methods, including electron microscopy and morphometric analysis, immunoblotting, and immunohistochemistry. (DOCX)

S1 Data Excel spreadsheet containing, in separate sheets, the underlying numerical data for figure panels 1B, 1D, 1E, 1F, 2A, 2B, 2C, 3A, 3B, 3C, 4A, 4C, 5A, 5B, 5C, 5D, 6A, 6B, 7B, 7C, 7D, 7E, 7F, 8A, 8B, 8C, 9A, 9B, 9C, 10A, 10C. (XLSX)

## Abbreviations

bHLH-Zip: basic helix–loop–helix leucine zipper
CC: corpus callosum
CNS: central nervous system
CV: conduction velocity
DTI: diffusion tensor imaging
EM: electron microscopy
FASN: fatty acid synthase
GFAP: glial fibrillary acidic protein
GSL: glycosphingolipid
HFD: high-fat diet
MAG: myelin-associated glycoprotein
MBP: myelin basic protein
MUFA: monounsaturated fatty acids
MRI: magnetic resonance imaging
ON: optic nerve
OPC: oligodendrocyte precursor cell
PC: phosphatidyl choline
PE: phosphatidyl ethanolamine
PI: phosphatidyl inositol
PLP: myelin proteolipid protein
PNS: peripheral nervous system
PS: phosphatidyl serine
PUFA: polyunsaturated fatty acids
SC: Schwann cells
SCAP: SREBP cleavage-activating protein
SD: standard diet
SFA: saturated fatty acids
SM: sphingomyelin
SQS: squalene synthase
SREBP: sterol regulatory element-binding protein
tdT: td-Tomato
WT: wild-type
