# Supplementary figures and images for: Oligodendroglial myelination requires astrocyte-derived lipids
Source: PLoS Biol. 2017 May 26;15(5):e1002605. doi: 10.1371/journal.pbio.1002605 (PMC5446120; doi:10.1371/journal.pbio.1002605)

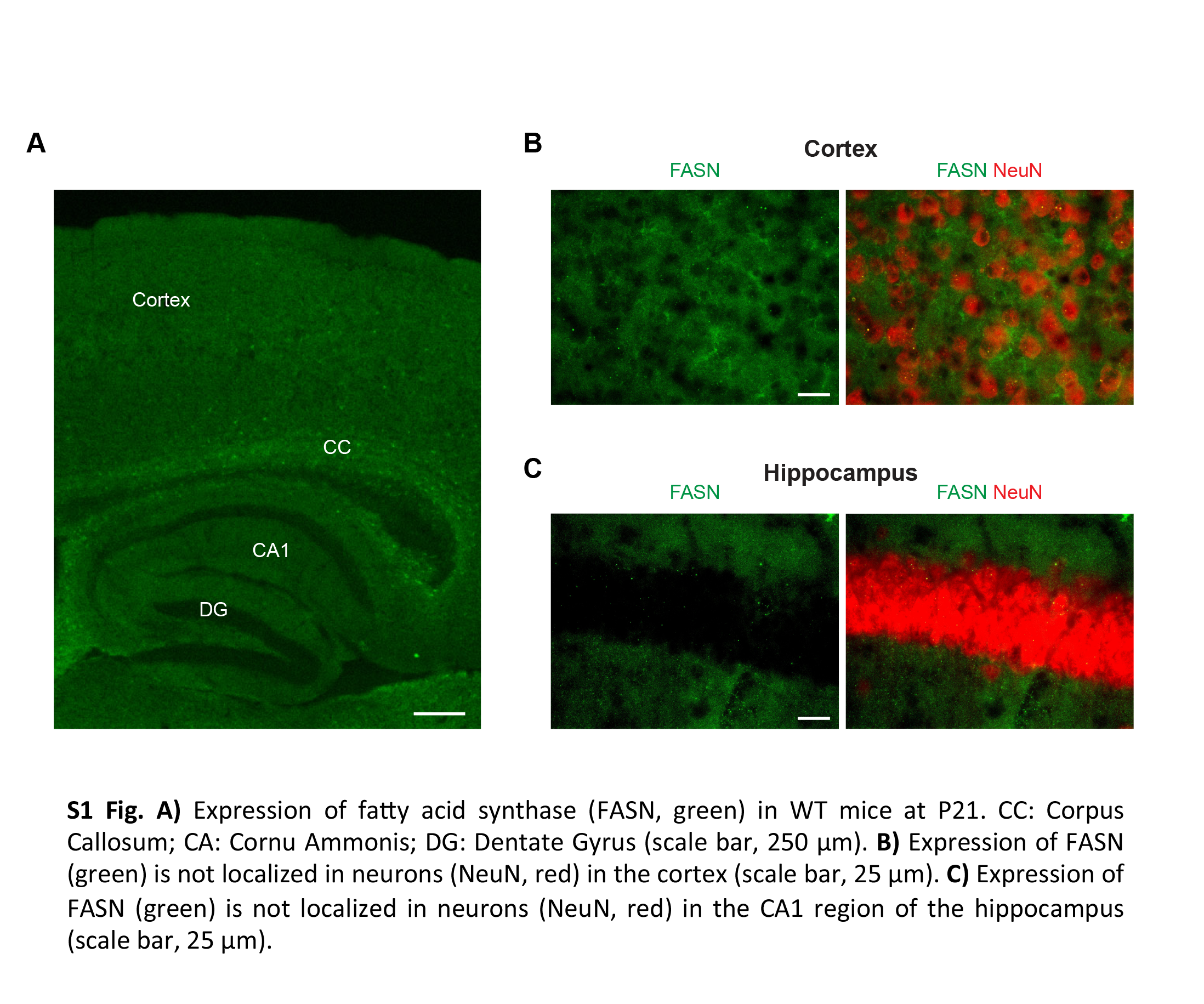

Supplement: S1 Fig — A) Expression of fatty acid synthase (FASN, green) in WT mice at P21. CC: Corpus Callosum; CA: Cornu Ammonis; DG: Dentate Gyrus (scale bar, 250 μm). B) Expression of FASN (green) is not localized in neurons (NeuN, red) in the cortex (scale bar, 25 μm). C) Expression of FASN (green) is not localized in neurons (NeuN, red) in the CA1 region of the hippocampus (scale bar, 25 μm). (TIF) [file pbio.1002605.s001.tif]

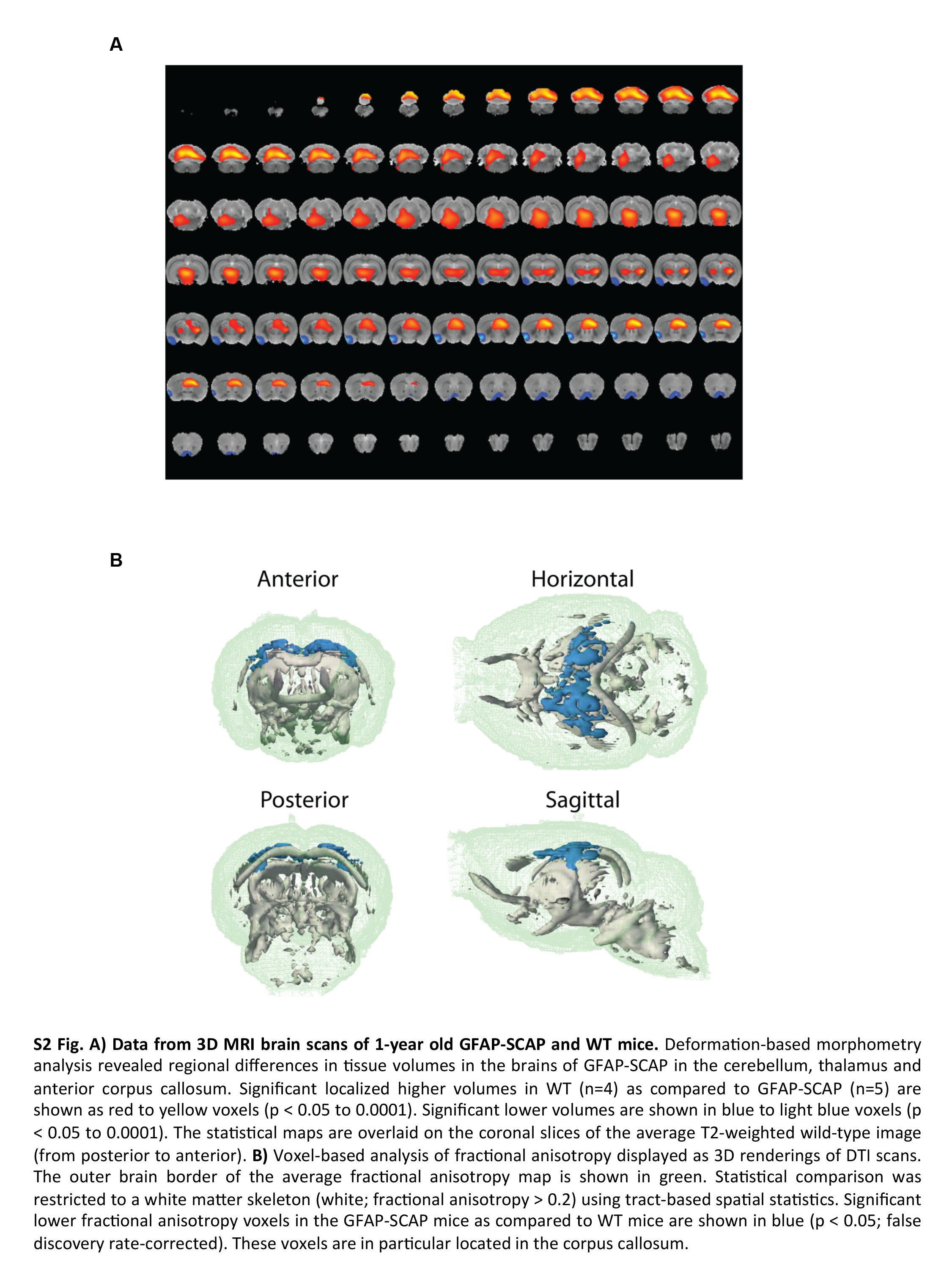

Supplement: S2 Fig — A) Data from 3D MRI brain scans of 1-year old GFAP-SCAP and WT mice. Deformation-based morphometry analysis revealed regional differences in tissue volumes in the brains of GFAP-SCAP in the cerebullum, thalamus, and anterior corpus callosum. Significant localized higher volumes in WT (n = 4) as compared to GFAP-SCAP (n = 5) are shown as red to yellow voxels (p < 0.05 to 0.0001). Significant lower volumes are shown in blue to light blue voxels (p < 0.05 to 0.0001). The statistical maps are overlaid on the coronal slices of the average T2-weighted wild-type image (from posterior to anterior). B) Voxel-based analysis of fractional anisotropy map is shown in green. Statistical comparison was restricted to a white matter skeleton (white; fractional anisotropy > 0.2) using tract-based spatial statistics. Significant lower fractional anisotropy voxels in the GFAP-SCAP mice as compared to WT mice are shown in blue (p < 0.05; false discovery rate-corrected). These voxels are in particular located in the corpus callosum. (TIF) [file pbio.1002605.s002.tif]

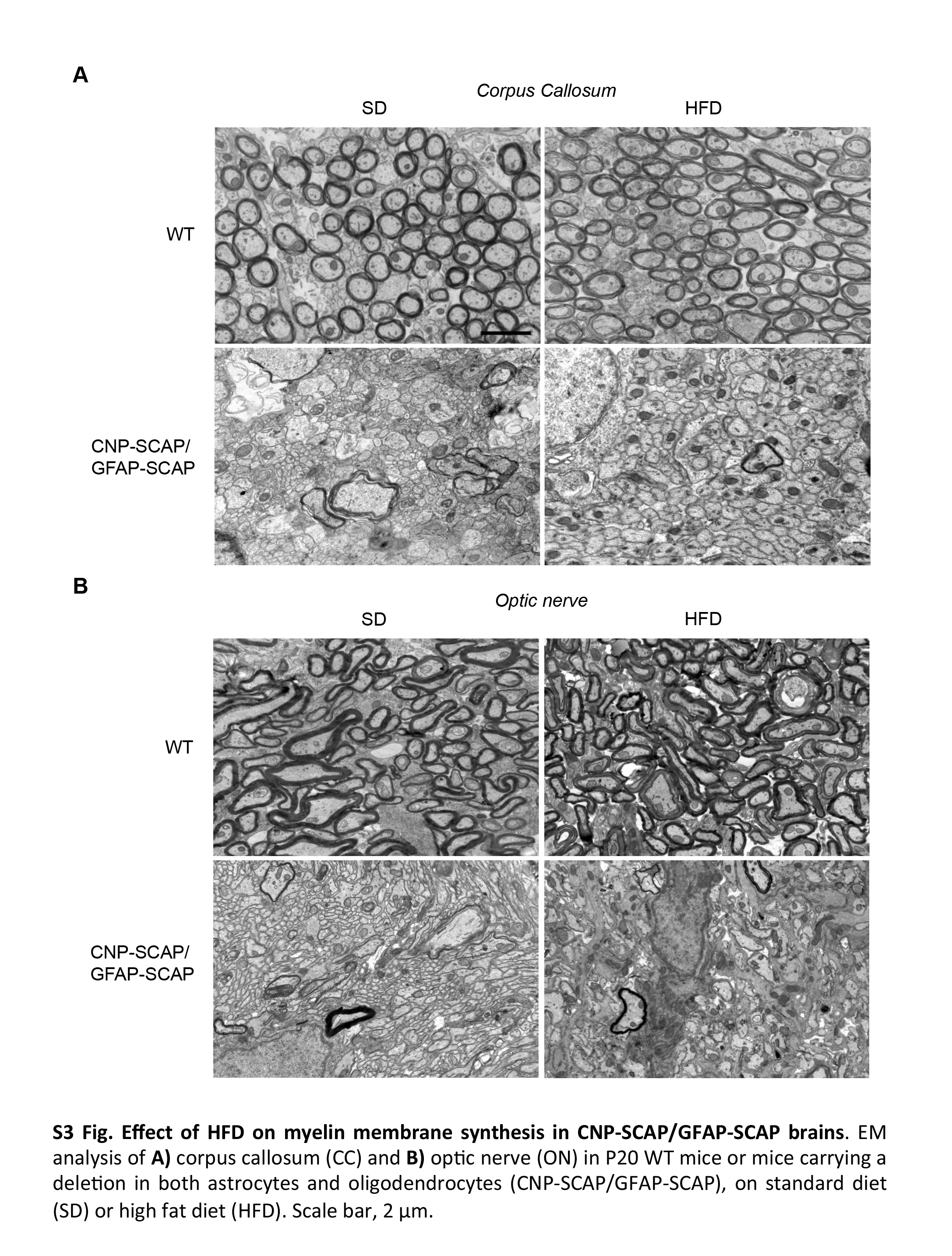

Supplement: S3 Fig — EM analysis of A) corpus callosum (CC) and B) optic nerve (ON) in P20 WT mice or mice carrying a deletion in both astrocytes and oligodendrocytes (CNP-SCAP/GFAP-SCAP), on standard diet (SD) or high fat diet (HFD). Scale bar, 2 μm. (TIF) [file pbio.1002605.s003.tif]

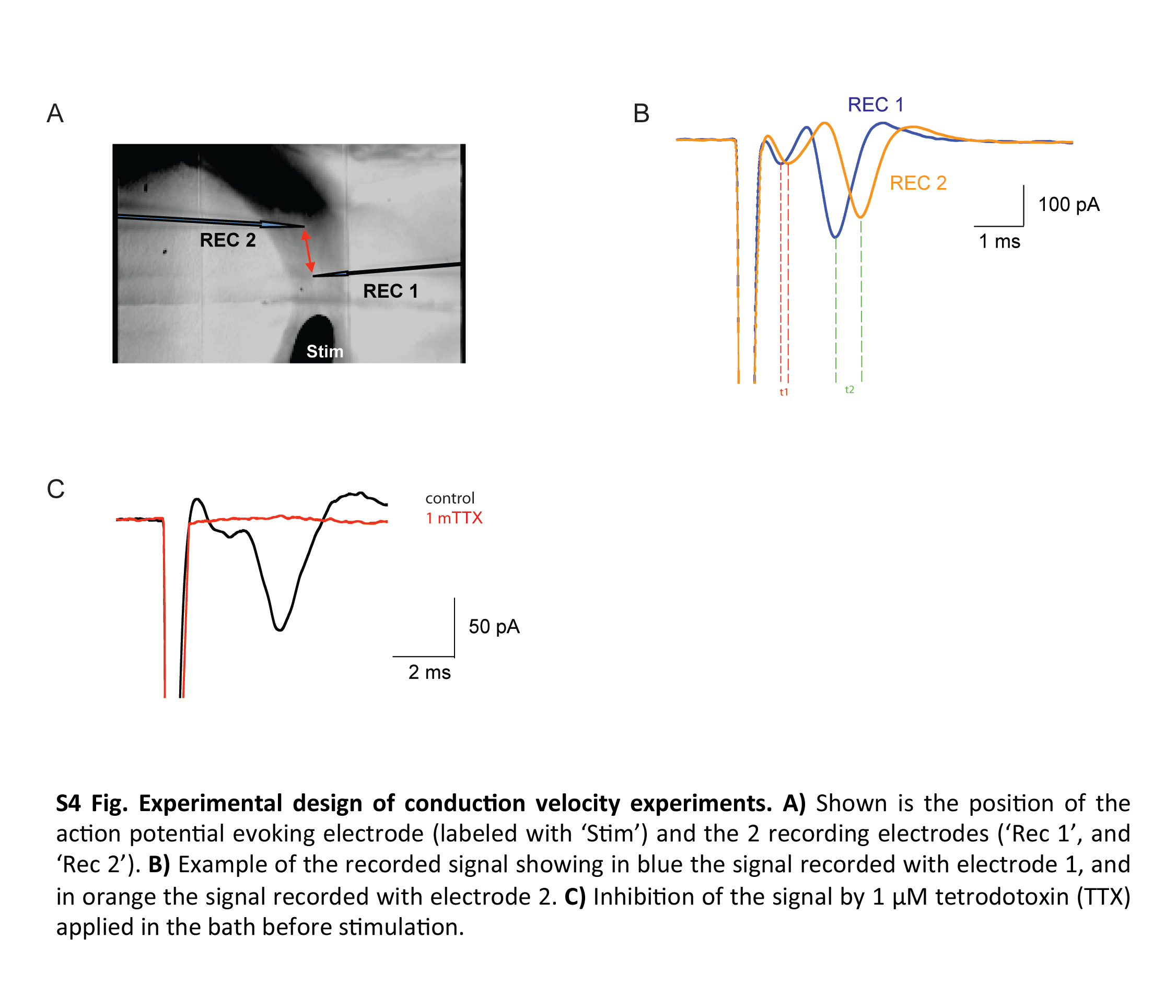

Supplement: S4 Fig — A) Shown is the position of the action potential evoking electrode (labelled with "Stim") and the 2 recording electrodes ("Rec 1," and "Rec 2"). B) Example of the recorded signal showing in blue the signal recorded with electrode 1, and in orange the signal recorded with electrode 2. C) Inhibition of the signal by 1 μm tetrodotoxin (TTX) applied in the bath before stimulation. (TIF) [file pbio.1002605.s004.tif]
