# Supplementary material for: Oligodendroglial myelination requires astrocyte-derived lipids
Source: PLoS Biol. 2017 May 26;15(5):e1002605. doi: 10.1371/journal.pbio.1002605 (PMC5446120; doi:10.1371/journal.pbio.1002605)
Supplement: S1 Text — (DOCX) [file pbio.1002605.s005.docx]

**Additional Methods**

**Electron Microscopy and Morphometric Analysis**

Animals under deep anesthesia were perfused transcardially with 20 ml of PBS 0.1 M, pH 7.4 containing 0.1% heparin followed by 100 mL of freshly prepared cold fixative solution composed of 4% paraformaldehyde in 0.1M PBS and 2% glutaraldehyde, pH 7.4. Brains were removed, postfixed overnight in fixative solution at 4°C, and cryoprotected with 30% sucrose for 2–3 days at 4°C. Brains were rapidly frozen in powdered dry ice and sliced into 50 μm-thick sagittal sections on a cryostat (-20°C). Free-floating sections were rinsed three times, dehydrated with ethanol and imbedded in Epon. Ultra-thin sections were subsequently cut, collected on formvar coated single slot grids, and stained with a 1% aqueous uranyl acetate solution for 20 min and subsequently for 1 min with lead citrate. Photographs were obtained by using a JEOL 1010 electron microscope. For each myelinated axon present the g-ratio was calculated by dividing the axonal diameter (defined by the inner limit of the myelin sheath) by the total fiber diameter (defined by the outer limit of the myelin sheath). Each group consisted of at least three mice.

**Immunoblotting**

Brains were dissected, separated from the spinal cord and medulla oblongata, cleaned from the meninges, and rapidly frozen in iso-pentane and kept at -80° C until used. Upon use, the tissue was homogenized in 5 mL of homogenisation buffer (5 mM of HEPES/NaOH pH 7.4, 0.32 M sucrose and complete EDTA-free protease inhibitory cocktail (Roche applied sciences), containing 0.016 U/mL of RNAse inhibitor, Invitrogen). Whole-brain extracts were mixed with SDS and heated to 90 °C for 5 min. Proteins were separated by SDS-PAGE in a Mini-Protean Electrophoresis system and electro-blotted overnight onto PVDF membranes. Membranes were probed with primary antibodies rabbit anti-Olig2 (1:500, Millipore), mouse anti-MBP (1:2000, Santa Cruz), goat anti-Mag2 (1:1000, Santa Cruz), mouse anti-CNP (1:1000, Sigma), mouse anti-NeuN (1:2000, Millipore), rabbit anti-GFAP (1:1,000, Dako) and mouse anti-ß-actin (Millipore, 1:1000); followed by an alkaline phosphatase-conjugated secondary antibody (1:1000, Dako). After reaction with the ECF substrate (GE Healthcare) membranes were scanned with an FLA instrument (Fujifilm). Quantification was done using the Quantity One software (Biorad).

**Immunohistochemistry**

Mice, under deep anesthesia, were perfused transcardially with 20 ml of PBS 0.1 M, pH 7.4 containing 0.1% heparin followed by 100 mL of freshly prepared cold fixative solution composed of 4% paraformaldehyde in 0.1M PBS, pH 7.4. Brains were removed, post-fixed overnight in fixative solution at 4°C, and cryoprotected with 30% sucrose for 2–3 days at 4 °C. Brains were rapidly frozen in powdered dry ice and sliced into 50 μm-thick sagittal sections on a cryostat (-20 °C). Free-floating sections were rinsed three times and stored in 0.1M PBS, pH 7.4, containing 0.1% NaN_3_ at 4°C or 0.1% anti-freeze solution at -20 °C until use. For immunostainings, sections were rinsed 4 times for 10 minutes in 0.1M PBS, permeabilized for 5 minutes in 0.1M PBS containing 0.5% Triton-X-100, blocked for 30 min in 0.1M PBS containing 0.1% Triton-X-100 and 10% normal goat serum, and subsequently incubated with primary antibody in incubating medium (0.1M PBS, 0.1% Triton-X-100 and 2% normal goat serum) with shaking for 72 hours at 4 °C. The sections were washed 4 times for 10 minutes in 0.1M PBS and incubated in incubating medium containing secondary antibody for 2 hours. Sections were washed 2 times with 0.1M PBS, 1 time with distilled water, and subsequently DNA counterstained in Hoechst reagent 33258 solution (Sigma) for 20 min, washed 2 times for 10 minutes with distilled water and mounted on glass slides. Primary antibodies were goat anti-Olig2 (1:30, Millipore), rabbit anti-Ki67 (1:300, Santa Cruz), mouse anti-CC1 (APC) (1:2000, Calbiochem), mouse anti-NeuN (1:2000, Millipore), mouse anti-GFAP (1:500, Sigma), rabbit-anti-FASN (1:1000, Abcam). Secondary antibody were Alexa fluor 488-conjugated goat anti-mouse (1:400), Alexa fluor 568-conjugated goat anti-mouse (1:400), Alexa fluor 488-conjugated goat anti-rabbit (1:400), Dyl488-conjugated donkey anti-goat (1:200), Dyl550-conjugated donkey anti-mouse (1:250) and Alexa fluor 647-conjugated donkey anti-rabbit (1:400) from Molecular probes. Sections were examined on a Leica DMi8 microscope, sampled with a Leiko DFC3000G camera under fluorescent illumination.
